# On fixed points and convergence results of sequences generated by uniformly convergent and point-wisely convergent sequences of operators in Menger probabilistic metric spaces

**DOI:** 10.1186/s40064-016-2057-0

**Published:** 2016-05-04

**Authors:** Manuel De la Sen, Asier Ibeas, Jorge Herrera

**Affiliations:** Institute of Research and Development of Processes IIDP, Faculty of Science and Technology, University of the Basque Country, PO Box 644, de Bilbao, Barrio Sarriena, 48940 Leioa, Bizkaia Spain; Department of Telecommunications and Systems Engineering, Faculty of Engineering, Universitat Autònoma de Barcelona (UAB), 08193 Bellaterra, Cerdanyola del Vallés, Barcelona Spain; Departamento de Ingeniería, Facultad de Ciencias Naturales e Ingeniería, Universidad de Bogotá Jorge Tadeo Lozano, 22 Street, No. 4-96, Mod. 7A, Bogotá, D.C. 110311 Colombia; Departamento de Ingeniería, Facultad de Ciencias Naturales e Ingeniería, Universidad de Bogotá Jorge Tadeo Lozano, 22 Street, No. 4-96, Mod. 7A, Bogotá, D.C. 110311 Colombia

**Keywords:** Strict contractions, Strict $\varphi$-contractions, Probabilistic metric spaces, Menger spaces, Triangular norms

## Abstract

In the framework of complete probabilistic Menger metric spaces, this paper investigates some relevant properties of convergence of sequences built through sequences of operators which are either uniformly convergent to a strict *k*-contractive operator, for some real constant *k* ∈ (0, 1), or which are strictly *k*-contractive and point-wisely convergent to a limit operator. Those properties are also reformulated for the case when either the sequence of operators or its limit are strict $\varphi$-contractions. The definitions of strict (*k* and $\varphi$) contractions are given in the context of probabilistic metric spaces, namely in particular, for the considered probability density function. A numerical illustrative example is discussed.

## Background

Fixed point theory is an important tool to investigate the convergence of sequences to limits and unique limits in metric spaces and normed spaces. See, for instance, Pap et al. (1996), Sehgal and Bharucha-Reid (1972), Schweizer and Sklar (1960), Eldred and Veeramani (2006), De la Sen (2010a, b), Choudhury et al. (2011, 2012), De la Sen and Karapinar (2014, 2015a, b), Beg et al. (2001), Roldan et al. (2014), Jleli et al. (2014), Roldán-Lopez-de-Hierro et al. (2015), Khan et al. (1984), Choudhury and Das (2008), Gopal et al. (2014), Takahashi (1970), Shimizu and Takahashi (1996), Kaewcharoen and Panyanak (2008), Karpagam and Agrawal (2009), Suzuki (2006), Di Bari et al. (2008), Rezapour et al. (2011), Derafshpour et al. (2010), Al-Thagafi and Shahzad (2009), Karpagam and Agrawal (2009), Dutta et al. (2009), Chang et al. (2001), Chen et al. (2012), Chen (2012), Berinde (2007), De la Sen et al. (2015) and the wide list of references cited in those papers. In particular, fixed point theory is also a relevant tool to investigate iterative schemes and stability theory of continuous-time and discrete-time dynamic systems, boundedness of the trajectory solutions, stability of equilibrium points, convergence to stable equilibrium points and the existence oscillatory solution trajectories. See, for instance, De la Sen (1999, 2010), Berinde (2007), De la Sen and Karapinar (2014), De la Sen et al. (2010, 2015), Marchenko (2014, 2015), Delasen (1983), Istratescu (1981) and references therein. The solution of some interesting stability and solution approximation problems in some integral and physical problems have been recently investigated in Eshkuvatov et al. (2015), Abdulla et al. (2015), Matinfar et al. (2015).

On the other hand, fixed point theory is, in particular, receiving important research attention in the framework of probabilistic metric spaces. See, for instance, Schweizer and Sklar (1960, 1983), Pap et al. (1996), Sehgal and Bharucha-Reid (1972), Choudhury et al. (2011, 2012), De la Sen and Karapinar (2015a), Beg et al. (2001) and references therein. Also, Menger probabilistic metric spaces are a special class of the wide class of probabilistic metric spaces which are endowed with a triangular norm, (Pap et al. 1996; Sehgal and Bharucha-Reid 1972; Choudhury et al. 2011; De la Sen and Karapinar 2015a, b; Choudhury and Das 2008; Gopal et al. 2014) and which are very useful in the context of fixed point theory since the triangular norm plays a close role to that of the norm in normed spaces. In probabilistic metric spaces, the deterministic notion of distance is considered to be probabilistic in the sense that, given any two points *x* and *y* of a metric space, a measure of the distance between them is a probabilistic metric *F*_*x*,*y*_(*t*), rather than the deterministic distance *d*(*x*, *y*), which is interpreted as the probability of the distance between *x* and *y* being less than *t* (*t* > 0), (Sehgal and Bharucha-Reid 1972).

Fixed point theorems in complete Menger spaces for probabilistic concepts of *B* and *C*-contractions can be found in Pap et al. (1996) together with a new notion of contraction, referred to as (*Ψ*, *C*)-contraction. Such a contraction was proved to be useful for multivalued mappings while it generalizes the previous concept of *C*-contraction. On the other hand, cyclic contractions on subsets of complete Menger spaces were discussed in Choudhury et al. (2011, 2012), De la Sen and Karapinar (2015a). Also, some types of contractions in complete probabilistic Menger spaces have been studied through the use of the so-called altering distances. See, for instance, Khan et al. (1984), De la Sen (2010) and references therein and more recent results in Mishra et al. (2015). Some general fixed point theorems have been very recently obtained in Gopal et al. (2014) for $\alpha - \psi$ contractive mappings in Menger probabilistic metric spaces. Also, a parallel background literature, related to results on best proximity points and fixed points in cyclic mappings in metric and Banach spaces as well as topics related to common fixed points, is exhaustive. See, for instance, Eldred and Veeramani (2006), De la Sen (2010), Takahashi (1970), Shimizu and Takahashi (1996), Kaewcharoen and Panyanak (2008), Karpagam and Agrawal (2009), Suzuki (2006), Di Bari et al. (2008), Rezapour et al. (2011), Derafshpour et al. (2010), Al-Thagafi and Shahzad (2009) and (Chen et al. 2012), Chen 2012) as well as references therein. On the other hand, fuzzy metric spaces have been investigated more recently and some ad -hoc versions of fixed point theorems have been obtained in that framework. See, for instance, Roldan et al. (2014), Jleli et al. (2014), Roldán-Lopez-de-Hierro et al. (2015) and some references therein. Recent research has been also focused on some basic convergence properties of the iterates in iterative schemes. For instance, a new averaged algorithm for finding a common fixed point of a countably infinite family of generalized k-strictly pseudocontractive multi-valued mappings is studied in Chidume and Opkala (2015) and the computational errors of iterated schemes in the set-valued case are investigated in Reich and Zaslavski (2015) and in Dinevari and Frigon (2015), in this last case under the support of graph theory. On the other hand, some variants of homopotopy methods together with Picard and Picard–Padé iterative methods to solve Michaelis–Menten equation have been investigated in Vazquez-Leal et al. (2015) while an iterative scheme being strongly convergent to a common fixed point of a countable family of strictly pseudo-contractive mappings has been focused on in Chamnarnpan et al. (2013). Such a fixed point is proved to be also a solution of variational inequality problem related to quadratic minimization problems. See also Chidume et al. (2015).

This paper investigates some properties of convergence of sequences being built through sequences of operators which are either uniformly convergent to a strict *k*-contractive operator, for some real constant *k* ∈ (0, 1), or which are strictly *k*-contractive and point-wisely convergent to a limit operator. The obtained properties are reformulated later on for the case when either the sequence of operators or its limit operator are strict $\varphi$-contractions. The appropriate definitions of strict (*k* and $\varphi$) contractions are given ad-hoc in the context of probabilistic metric spaces, namely, for the considered probability density function.

Denote ${\varvec{R}}_{+} = \{z \in {\varvec{R}}: z > 0\}$, ${\varvec{R}}_{0+} = {\varvec{R}}_{+} \cup \{0\}$, ${\varvec{Z}}_{+} = \left\{{z \in {\varvec{Z}} : z > 0} \right\}$, ${\varvec{Z}}_{0+} = {\varvec{Z}}_{+} \cup \{0\}$, $\bar{n} = \{1, 2, \ldots,n\}$, and denote also by ***L***, the set of distance distribution functions $H:{\varvec{R}} \to [0, 1]$, (Schweizer and Sklar 1983), which are non-decreasing and left continuous such that *H*(0) = 0 and $sup_{t \in {\varvec{R}}} H(t) = 1$. Let *X* be a nonempty set and let the probabilistic metric (or distance) $F: X \times X \to L$ a symmetric mapping from *X* × *X*, where *X* is an abstract set, to the set of distance distribution functions *L* of the form $H:{\varvec{R}} \to [0, 1]$ which are functions of elements $F_{x,y}$ for every $(x, y) \in X \times X$. Then, the ordered pair $(X, F)$ is a probabilistic metric space (PM) (Pap et al. 1996; Sehgal and Bharucha-Reid 1972; Chang et al. 2001) if

1 $$\begin{aligned} & 1. \quad \forall x,y \in X \left({\left({F_{x,y} (t) = 1; \forall t \in {\varvec{R}}_{+}} \right) \Leftrightarrow \left({x = y} \right)} \right) \\ & 2. \quad F_{x,y} (t) = F_{y,x} (t); \forall x,y \in X,\; \forall t \in {\varvec{R}} \\ & 3. \quad \forall x,y,z \in X; \forall t_{1},t_{2} \in {\varvec{R}}_{+} \left((F_{x,y} (t_{1}) = F_{y,z} (t_{2}) = 1) \Rightarrow \left(F_{x,z} (t_{1} + t_{2}) = 1\right) \right) \end{aligned}$$

A particular distance distribution function *F*_*x*,*y*_ ∈ *L* is a probabilistic metric (or distance) which takes values *F*_*x*,*y*_(*t*) identified with a probability distance density function $H:{\varvec{R}} \to [0, 1]$ in the set of all the distance distribution functions ***L***.

A Menger PM-space is a triplet $(X,F,\varDelta)$, where $(X, F)$ is a PM-space which satisfies:

2 $$F_{x,y} \left({t_{1} + t_{2}} \right) \ge \varDelta \left({F_{x,z} \left({t_{1}} \right), F_{z,y} \left({t_{2}} \right)} \right);\quad \forall x, y, z \in X,\;\forall t_{1},t_{2} \in {\varvec{R}}_{0+}$$

under $\varDelta: [0,1] \times [0, 1] \to [0,1]$ is a *t*-norm (or triangular norm) belonging to the set ***T*** of *t*-norms which satisfy the properties:

1. *Δ*(*a*, 1) = *a*
2. *Δ*(*a*, *b*) = *Δ*(*b*, *a*)
3. *Δ*(*c*, *d*) ≥ *Δ*(*a*, *b*) if *c* ≥ *a*, *d* ≥ *b*
4. 3 $$\varDelta (\varDelta(a,b),c) = \varDelta (a,\varDelta (b,c))$$

A property which follows from the above ones is *Δ*(*a*, 0) = 0 for $a \in [0,1]$. Typical continuous *t*-norms are the minimum *t*-norm defined by *Δ*_*M*_(*a*, *b*) = *min* (*a*, *b*), the product *t*-norm defined by *Δ*_*P*_(*a*, *b*) = *a*.*b* and the Lukasiewicz (or nilpotent-minimum) *t*-norm defined by *Δ*_*L*_(*a*, *b*) = *max* (*a* + *b* − 1, 0) which are related by the inequalities *Δ*_*L*_ ≤ *Δ*_*P*_ ≤ *Δ*_*M*_.

The (probabilistic) diameter of a subset *A* of *X* is a function from ${\varvec{R}}_{0+}$ to [0, 1] defined by $D_{A} (z) = sup_{t < z} inf_{x, y \in A} F_{x,y} (t)$ and *A* is probabilistically bounded if $D_{A}^{p} = sup_{z \in {\varvec{R}}_{+}} D_{A} (z) = 1$ [$D_{A}^{p}$ can be defined equivalently as *lim*_*z*→∞_*D*_*A*_(*z*)], probabilistically semibounded if 0 < $D_{A}^{p}$ < 1 and probabilistically unbounded if $D_{A}^{p}$ = 0, (Schweizer and Sklar 1983; Pap et al. 1996). The diameter of a subset *A* ⊂ *X* in the *PM*-space (*X*, *F*), induced by a metric space (*X*, *d*), refers to maximum real interval measure, where the argument of the probabilistic metric is unity.

### Notation and some basic definitions

- ***Z***, ***Z***_+_, ***R*** and ***R***_+_ are the sets of integers, positive integers, real and positive real numbers, and ${\varvec{Z}}_{0+} = {\varvec{Z}}_{+} {\backslash}\{0\}$ and ${\varvec{R}}_{0+} = {\varvec{R}}_{+} {\backslash}\{0\}$, respectively.
- $\bar{m} = \{1,2,\ldots,m\}$ for any given $m \in {\varvec{Z}}_{+}$.
- $\left\{T_{n}\right\} \to T$ denotes point-wise convergence of the sequence of operators {*T*_*n*_} to the operator *T*, where
  $$T_{n},T : X \to X;\forall n \in {\varvec{Z}}_{+}.$$
- $\{T_{n}\} \rightrightarrows T$ denotes uniform convergence of the sequence of operators {*T*_*n*_} to the operator *T*.
- The symbol “${\neg}$” denotes logic negation.
- A probabilistic distance is a mapping ${\varvec{F}}: X \times X \to \varDelta_{\varvec{F}}$, where *X* is a nonempty abstract set represented by *F*_*x*,*y*_ for each (*x*, *y*) ∈ *X* × *X* and $\varDelta_{\varvec{F}}$ is a set of distribution functions such that $F \in \varDelta_{\varvec{F}}$ is a mapping $F:{\varvec{R}} \to {\varvec{R}}_{0+}$ which is non-decreasing and left-continuous with $inf_{t \in {\varvec{R}}} F (t) = 0$ and $sup_{t \in {\varvec{R}}} F (t) = 1$.
- The ordered pair $(X, {\varvec{F}})$ is a probabilistic metric (PM) space if for any $x, y, z \in X$ and all $t,s \in {\varvec{R}}_{+}$ the following conditions hold, (Schweizer and Sklar 1983):

4 $$ \begin{aligned} &1. \quad F_{x,y} (t) = H(t) \Leftrightarrow x = y\\ & \qquad \text{where}\; H \in \varDelta_{\varvec{F}}\;\text{is\;defined\;by}\;H (t) = \left\{\begin{array}{ll} 0& \quad if\;t \le 0 \\ 1 & \quad if\; t > 0 \\ \end{array} \right.;\\ & 2. \quad F_{x,y} (t) = F_{y,x} (t);\\ & 3. \quad if\;Fx,y(t) = 1\;and\;Fy,z(s) = 1\;then\;Fx,z(t + s) = 1.\end{aligned} $$

- The triplet $(X, {\varvec{F}},\varDelta)$ is a Menger space, where $(X, {\varvec{F}})$ is a PM-space and $\varDelta : [0,1] \times [0,1] \to [0,1]$ is a triangular norm, which satisfies the inequality $F_{x,z} (t + s) \ge \varDelta \left({F_{x,y} (t),F_{y,z} (s)} \right);\forall x, y, z \in X; \; \forall t,s \in {\varvec{R}}_{+}$.
- $\varDelta_{M} : [0,1] \times [0,1] \to [0,1]$ is the minimum triangular norm defined by $\varDelta_{M} (a,b) = min (a,b)$.
- A sequence {*x*_*n*_} ⊆ *X* in a probabilistic space $(X, {\varvec{F}})$ is said to be:
  1. almost surely (a.s.) convergent to a point *x* ∈ *X*, denoted by {*x*_*n*_} → *x* (a.s.), if for every $\varepsilon \in {\varvec{R}}_{+}$ and $\lambda \in (0,1)$, there exists some $N = N(\varepsilon,\lambda) \in {\varvec{Z}}_{0+}$ such that:
    $$F_{x_{n},x} (\varepsilon) > 1 - \lambda;\quad \forall n (\in {\varvec{Z}}_{0+}) \ge N,$$
  2. Probabilistically convergent (or, equivalently, it converges in probability) to a point *x* ∈ *X*, denoted by {*x*_*n*_} → *x*, if the probabilistic $(X,{\varvec{F}})$ is induced by a metric space (*X*, *d*) and for every $\varepsilon \in {\varvec{R}}_{+}$ and $\lambda \in (0,1)$ there exists some $N = N(\varepsilon,\lambda) \in {\varvec{Z}}_{0+}$ such that:
    $$F_{x_{n},x} \left({d (x_{n},x) - \varepsilon} \right) \le 1 - \lambda;\quad \forall n\left({\in {\varvec{Z}}_{0+}} \right) \ge N,$$
  3. Cauchy if for every $\varepsilon \in {\varvec{R}}_{+}$ and $\lambda \in (0,1)$, there exists some $N = N(\varepsilon,\lambda) \in {\varvec{Z}}_{0+}$ such that
    $$F_{{x_{n},x_{m}}} (\varepsilon) > 1 - \lambda;\quad \forall n,m \left({\in {\varvec{Z}}_{0+}} \right) \ge N.$$
  A good treaty on almost sure convergence and martingale approaches has been given in Stout (1974). Note that a PM-space $(X, {\varvec{F}})$ is complete if every Cauchy sequence is almost surely convergent.

#### **Lemma 1**

*Consider a PM-space*$(X, {\varvec{F}})$* and a sequence*$\left\{{x_{n}} \right\} \subset X$.* Then, the following properties hold:*

(i) {*x*_*n*_} → *x a.s. if and only if*$lim_{n \to \infty} F_{{x_{n + \ell},x}} (\varepsilon) =lim_{n,m \to \infty} F_{{x_{n + m},x}} (\varepsilon) = 1;\;\forall \ell \in {\varvec{Z}}_{0+},\;\forall \varepsilon \in {\varvec{R}}_{+}.$
(ii) {*x*_*n*_} → *x if and only if*$lim_{n \to \infty} F_{x_{n},x} \left({d\left(x_{n},x \right) - \varepsilon} \right) = 0;\forall \varepsilon \in {\varvec{R}}_{+}$*, where*$\left({X,{\varvec{F}}} \right)$* is induced by a metric space *(*X*, *d*).
(iii) *If* {*x*_*n*_} → *x a.s. and, furthermore, the PM space*$(X, {\varvec{F}})$* is induced by a metric space* (*X*, *d*)* then *{*x*_*n*_} → *x*.
(iv) *If *{*x*_*n*_} → *x a.s. then *{*x*_*n*_}* is Cauchy. The converse is true if*$(X, {\varvec{F}})$* is complete*.
(v) *Assume that the PM space*$(X, {\varvec{F}})$* is induced by a metric space* (*X*, *d*)* and that the distribution function is*$F_{x,y} (t) = H (t - d(x,y)); \forall x, y \in X.$* Then, almost sure convergence and convergence in probability are equivalent for any sequence* {*x*_*n*_} ⊂ *X*.

#### *Proof*

Since {*x*_*n*_} → *x* (a.s.), then, for every given $\varepsilon \in {\varvec{R}}_{+}$ and $\lambda \in (0,1)$, there exists some $N = N(\varepsilon,\lambda) \in {\varvec{Z}}_{0+}$ such that $F_{x_{n},x} (\varepsilon) > 1 - \lambda; \forall n\left({\in {\varvec{Z}}_{0+}} \right) \ge N$. Then, ${lim\;inf}_{n \to \infty} \left({{lim\;inf}_{{\varepsilon \to 0^{+}}} F_{x_{n},x} (\varepsilon)} \right) > 1 - \lambda$ for any given $\lambda \in (0,1)$ since $F_{x_{n},x} (\varepsilon)$ is left-continuous and non-decreasing. This leads to the existence of the limit $lim_{n \to \infty} F_{{x_{n + j},x}} (\varepsilon) = lim_{n \to \infty} F_{x_{n},x} \left({0^{+}} \right) = 1,\forall \varepsilon \in {\varvec{R}}_{+},\forall j \in {\varvec{Z}}_{0+}$. Conversely, since $lim_{n \to \infty} F_{x_{n},x} (\varepsilon) = 1$, there is a ball $B_{N} (x,\varepsilon,\lambda)$ in the PM space $(X, {\varvec{F}})$ which contains *x*_*n*_ for all $n\left({\in {\varvec{Z}}_{0+}} \right) \ge N$ and some *N* = *N*(*ɛ*, *λ*) so that $F_{x_{n},x} (\varepsilon) > 1 - \lambda;\forall n \ge N$. Property (i) has been proved.

On the other hand if {*x*_*n*_} → *x*, if for every given $\varepsilon \in {\varvec{R}}_{+}$ and $\lambda \in (0,1)$ there exists some $N = N(\varepsilon,\lambda) \in {\varvec{Z}}_{0+}$ such that $F_{x_{n},x} \left({d (x_{n},x) - \varepsilon} \right) \le 1 - \lambda;\forall n\left({\in {\varvec{Z}}_{0+}} \right) \ge N$. The condition for *d*(*x*_*n*_, *x*) ≤ *ɛ* holds trivially since $F_{x_{n},x} \left(0 \right) = 0,\forall n \in {\varvec{Z}}_{0+}$ so that we discuss the case *d*(*x*_*n*_, *x*) > *ɛ*. By taking *λ* = 1 – *δ* and arbitrary *δ* ∈ (0, 1), one gets ${lim\;sup}_{n \to \infty} F_{x_{n},x} \left({d (x_{n},x) - \varepsilon} \right) \le \delta$ so that the limit $lim_{n \to \infty} F_{x_{n},x} \left({d (x_{n},x) - \varepsilon} \right) = 0$ exists for any $\varepsilon \in {\varvec{R}}_{+}$ if {*x*_*n*_} → *x*. The converse is proved closely to the proof of its counterpart in Proposition (i) by defining a ball $B_{N} \left({x,d (x_{n},x) - \varepsilon,\lambda} \right)$ in the PM space $(X, {\varvec{F}})$ which contains *x*_*n*_ for all $n\left({\in {\varvec{Z}}_{0+}} \right) \ge N$. Property (ii) has been proved.

To prove Property (iii), note from Property (i) that {*x*_*n*_} → *x* a.s. if and only if $lim_{n \to \infty} F_{x_{n},x} \left({0^{+}} \right) = 1$ and the sequence in the metric space (*X*, *d*) satisfies *d*(*x*_*n*_, *x*) → 0 as *n* → ∞. Assume that {*x*_*n*_} → *x* fails, so that ${lim\;inf}_{n \to \infty} F_{x_{n},x} \left({d(x_{n},x) - \varepsilon} \right) > 0$ for some $\varepsilon \in {\varvec{R}}_{+}$, and proceed by contradiction by assuming that there exists some $\varepsilon \in {\varvec{R}}_{+}$ such that ${lim\;inf}_{n \to \infty} \left({d(x_{n},x) - \varepsilon} \right) > lim_{n \to \infty} d(x_{n},x) = 0$. But this leads to the contradiction ${lim\;inf}_{n \to \infty} F_{x_{n},x} \left({- t} \right) > 0$ for some $t \in {\varvec{R}}_{+}$.

The proof of Property (iv) is given as follows. Now, if {*x*_*n*_} → *x* a.s. then for any given $\varepsilon \in {\varvec{R}}_{+}$ and $\lambda \in (0,1)$:

$$\mathop {lim}\limits_{n \to \infty} F_{{x_{n + \ell},x}} \left({\varepsilon/2} \right) = \mathop {lim}\limits_{n \to \infty} F_{x_{n},x} \left({\varepsilon/2} \right) = 1;\quad \forall \ell \in {\varvec{Z}}_{+}.$$

Then, by the third property of (5), one gets $lim_{n \to \infty} F_{{x_{n + \ell},x_{n}}} (\varepsilon) = 1; \forall \ell \in {\varvec{Z}}_{0+}$. Assume that {*x*_*n*_} is not Cauchy. Thus, for some given $\varepsilon \in {\varvec{R}}_{+}$ and $\lambda \in (0,1)$, and any $N \in {\varvec{Z}}_{0+}$, there is a subsequence $\left\{{n_{k}} \right\} \subseteq {\varvec{Z}}_{0+}$ with *m*_*k*_ > *n*_*k*_ ≥ *N* such that $1 - \lambda \ge lim_{k \to \infty} F_{{x_{{n_{k},}} x_{{m_{k}}}}} (\varepsilon)$ and then ${lim\;sup}_{k \to \infty} F_{{x_{{n_{k},}} x_{{m_{k}}}}} (\varepsilon) < 1$ but implies that $lim_{n \to \infty} F_{{x_{n + \ell},x_{n}}} (\varepsilon) < 1$, a contradiction. Then, if $lim_{n \to \infty} F_{{x_{n + \ell},x_{n}}} (\varepsilon) = 1$, and by Property (i), if {*x*_*n*_} → *x* a.s., then {*x*_*n*_} is Cauchy. The converse is true if the metric space is complete from the definition of completeness. Property (iv) has been proved.

To prove Property (v), note that $F_{{x_{n},x}} (\varepsilon) = H \left({\varepsilon - d\left({x_{n},x} \right)} \right)$. If {*x*_*n*_} → *x* then $lim_{n \to \infty} F_{x_{n},x} \left({d(x_{n},x) - \varepsilon} \right) = 0$, and then $lim_{n \to \infty} F_{x_{n},x} \left({\varepsilon - d(x_{n},x)} \right) = 1$ for any $\varepsilon \in {\varvec{R}}_{+}$ so that from Property (i), {*x*_*n*_} → *x* a.s.. The converse, that is,{*x*_*n*_} → *x* (a.s.) ⇒ {*x*_*n*_} → *x* if $\left({X,{\varvec{F}}} \right)$ is induced by (*X*, *d*) is Property (iii), already proved. Thus, if $\left({X,{\varvec{F}}} \right)$ is induced by $\left({X,d} \right)$ then {*x*_*n*_} → *x* a.s. $\Leftrightarrow$ {*x*_*n*_} → *x*.$\square$

#### *Example 1*

Consider the probabilistic metric $\left({{\varvec{Z}}_{+},{\varvec{F}}} \right)$ space with probability density function

$$F_{ij} (t) = \left\{\begin{array}{ll} 1 &\quad if\;(j-1)/i < t \le j/i \\ 0&\quad otherwise \\ \end{array} \right.;\quad \forall i,j \in {\varvec{Z}}_{+}.$$

Then, the sequence of probability densities $\left\{F_{ij}(t)\right\} = \left\{F_{11}(t), F_{21}(t), F_{22}(t),F_{31}(t), F_{32}(t), F_{33}(t),\ldots \right\}$ defined for all $i,j (\le i) \in {\varvec{Z}}_{+}$ and *t* ∈ (0, 1) does not converge to one as *i*, *j* → ∞, so that it does not converge almost surely since *lim* _*n*→∞_*F*_(*n*−1)/*n*,1_(1) = 1, since the argument *t* = *t*(*n*) satisfies, $t \in \left({\frac{n - 1}{n},1} \right]$, and $lim_{n \to \infty} F_{j/n,(n - 1)/n} (t) = lim_{j({\le}n - 1),n \to \infty} F_{j/n,(n - 1)/n} (t) = 0;\forall j (\le n - 1) \in {\varvec{Z}}_{+},t \in [0, 1)$.

However, $\left\{{F_{n, n + 1} (t)} \right\}$ converges in probability as *n* → ∞; $\forall t \in {\varvec{R}}_{+}$ since

$$\begin{aligned}{\mathop{lim}\limits_{n \to \infty}} \left|1 - F_{n, n + 1} (t) \right| &= \left\{\begin{array}{ll} 0& \quad if \left[\left(1 < t \le 1 + {\mathop{lim}\limits_{n \to \infty}} 1/n\right) \vee (t = 0) \right]\\ 1& \quad if \; t \in (0,1] \\ \end{array} \right. \\ &= \left\{\begin{array}{ll} 0& \quad if\; t = 0\\ 1& \quad if\; t \in (0,1] \\ \end{array} \right.\end{aligned}$$

## Main results concerning the uniform convergence of operators

The concept of (probabilistic) *k*-contraction follows:

### **Definition 1**

Let $\left({X,{\varvec{F}}} \right)$ be a PM-space. Then, $T : X \to X$ is *k*-contractive (or a *k*-contraction) if $F_{Tx,Ty} \left({k t} \right) \ge F_{x,y} (t);\forall x, y \in X,\forall t \in {\varvec{R}}_{+}$ for some real constant $k \in \left({0,1} \right)$.

A wider class of contractive (in a weaker sense) operators $T : X \to X$ is that which satisfies $F_{Tx,Ty} (t) > F_{x,y} (t)$, ∀*x*, *y*(≠*x*) ∈ *X*, $\forall t \in {\varvec{R}}_{+}$.

### **Proposition 1**

*Let *$(X, {\varvec{F}})$* be aPM-space. If *$T: X \to X$* is a -contraction then it is also a weak contraction. The converse is not true.k*

### *Proof*

Note that if $T: X \to X$ is a *k*-contraction then

$$F_{Tx,Ty} (t) \ge F_{x,y} \left({k^{-1} t} \right) \ge F_{x,y} (t)$$

since $F:{\varvec{R}} \to {\varvec{R}}_{0+}$ which is non-decreasing and left-continuous. Furthermore, if $x \ne y$ and $F_{x,y} \left({t_{1}} \right) < 1$ for some $t_{1} \in {\varvec{R}}_{+}$ then the last above inequality has to be strict for all $t \in {\varvec{R}}_{+}$, i.e. $F_{Tx,Ty} (t) > F_{x,y} (t);\forall t \in {\varvec{R}}_{+}$. Otherwise, we would have the following contradiction for *t* = *t*_1_:

$$1 = \mathop {lim}\limits_{n \to \infty} F_{{T^{n} x,T^{n} y}} (t) \ge F_{x,y} \left({k^{- n} t} \right) = F_{x,y} \left({+ \infty^{-}} \right) = F_{x,y} (t) > F_{x,y} \left({t_{1}} \right);\quad\forall t \in {\varvec{R}}_{+}$$

Since $F_{x,y} \left({t_{1}} \right) > F_{x,y} \left({t_{1}} \right)$ is impossible. On the other hand, it always exist such a $t_{1} \in {\varvec{R}}_{+}$ such that $F_{x,y} \left({t_{1}} \right) < 1;\forall x,y\left({\ne x} \right) \in X$. Otherwise, *x* = *y* from the first condition of (4).

As a result, if $T : X \to X$ is a *k*-contraction and $x \ne y$ then $F_{Tx,Ty} (t) > F_{x,y} (t);\forall x,y\left({\ne x} \right) \in X$. Thus, if $T : X \to X$ is a *k*-contraction then it is also contractive and we have specifically proved that for any real constant $k \in \left({0,1} \right)$ and any $x,y\left({\ne x} \right) \in X$:

$$F_{Tx,Ty} (t) \ge F_{x,y} \left({k^{-1} t} \right) \Rightarrow F_{Tx,Ty} (t) > F_{x,y} (t)$$

To prove that the inverse is not true, it suffices to prove that for some $k \in \left({0,1} \right)$ and any $x,y\left({\ne x} \right) \in X$, the logic implication below fails:

$$F_{Tx,Ty} (t) > F_{x,y} (t) \Rightarrow F_{Tx,Ty} (t) \ge F_{x,y} \left({k^{-1} t} \right)$$

If the above implication were true then the contradiction 1 > 1 would follow from:

$$1 \ge \mathop {lim}\limits_{n \to \infty} F_{{T^{n + 1}x, T^{n + 1} y}} (t) > \mathop {lim}\limits_{n \to \infty} F_{{T^{n} x,T^{n} y}} (t) \ge \mathop {lim}\limits_{n \to \infty} F_{x,y} \left({k^{- n} t} \right) = F_{x,y} \left({+ \infty^{-}} \right) = 1.$$

$$\square$$

The result below refers to the case of uniform convergence of the sequence $\{T_{n}\}$ on *X* to a strict *k*-contractive operator on *X* in the framework of a complete Menger space $\left({X, {\varvec{F}},\varDelta} \right)$ :

### **Theorem 1**

*Let *$\left({X, {\varvec{F}},\varDelta_{M}} \right)$* be a complete Menger space and let *$\{T_{n}\}$* be a sequence of operators *$T_{n} :X \to X$*, such that *$$F_{T_{n}} = \left\{{x_{n}^{*}} \right\},\forall n \in {\varvec{Z}}_{+},\left\{{T_{n}} \right\} \begin{array}{*{20}c} {_{\to}} \\ {^{\to}} \\ \end{array} \{T\}$$* a.s. and *$T: X \to X$* is a (strict) -contraction with kF*_*T*_ = {x^*^}.* Then, the following properties hold:*

(i) $$\begin{aligned} & lim_{n \to \infty} F_{{x_{n}^{*},x^{*}}} (t) = lim_{n \to \infty} F_{{x_{n}^{*},x^{*}}} \left({0^{+}} \right) = 1;\quad\forall t \in {\varvec{R}}_{+},\\ &\left\{{x_{n}^{*}} \right\} \to x^{*}\;{\rm a.s.\;and}\;T^{m} x_{n}^{*} \to x^{*}\;{\rm a.s.\;as}\; n, m \to \infty,\\ \end{aligned}$$
(ii) $$\begin{aligned} & lim_{m,n \to \infty} F_{{T^{m} x_{n}^{*},x^{*}}} (t) = lim_{n \to \infty} F_{{T^{\ell} x_{n}^{*},x^{*}}} (t) = lim_{m,n \to \infty} F_{{T^{m} x_{n}^{*},x^{*}}} \left({0^{+}} \right) = 1;\quad\forall \ell \in {\varvec{Z}}_{0+},\forall t \in {\varvec{R}}_{+},\\ & lim_{n \to \infty} F_{{x_{n}^{*},x^{*}}} (t) = lim_{n \to \infty} F_{{x_{n}^{*},x^{*}}} \left({0^{+}} \right) = 1;\quad\forall t \in {\varvec{R}}_{+}, \quad \left\{{T^{m} x_{n}^{*}} \right\} \to x^{*},\quad\forall m \in {\varvec{Z}}_{0+}. \\ \end{aligned}$$

### *Proof*

Fix any $t \in {\varvec{R}}_{+}$, $\gamma \in (0,1)$ and choose a natural number $N = N\left({\gamma,t} \right)$ such that *n* ≥ *N* implies

$$F_{{Tx,T_{n} x}} (t) \ge 1 - \gamma;\quad\forall x \in X,\;\forall m \in {\varvec{Z}}_{0+}$$

$\forall m \in {\varvec{Z}}_{+}$, since $$\left\{{T_{n}} \right\}\begin{array}{*{20}c} {_{\to}} \\ {^{\to}} \\ \end{array} \left\{{T} \right\}$$ a.s., ∀*x* ∈ *X* and, in particular, the above inequality holds for the fixed point x^*^of $T: X \to X$, where $k \in (0,1)$ is the contraction coefficient. Also, since {*x*_n_^*^}, {*Tx*_n_^*^} ⊂ *X*, x^*^ = Tx^*^ ∈ *X*, *x*_n_^*^ = *T*_*n*_*x*_n_^*^ ∈ *X*, $T: X \to X$ is a *k*-contraction and $F \in \varDelta_{\varvec{F}}$ is non-decreasing and left-continuous everywhere in its definition domain, one has for all *n* ≥ *N* and all $t \in {\varvec{R}}_{+}$ that:

5 $$\begin{aligned} F_{{x_{n}^{*},x^{*}}} (t) & = F_{{T_{n} x_{n}^{*},Tx^{*}}} (t) \\ &\ge \varDelta_{M} \left({F_{{Tx^{*},Tx_{n}^{*}}} \left({\eta t} \right),F_{{Tx_{n}^{*},T_{n} x_{n}^{*}}} \left({\left({1 - \eta} \right) t} \right)} \right) \\ &\ge \varDelta_{M} \left({F_{{x_{n}^{*},x^{*}}} \left({k^{-1} \eta t} \right),1 - \lambda} \right) \\ &> min \left({F_{{x_{n}^{*},x^{*}}} \left({k^{-1} \eta t} \right),1 - \lambda} \right) \\ \end{aligned}$$

for any given real constant $\eta \in \left({0,1} \right)$ since $F_{{Tx_{n}^{*},T_{n} x_{n}^{*}}} \left({t} \right) \ge 1 - \gamma > 1 - \lambda$; ∀*x* ∈ *X*, ∀*t* ∈ ***R***_+_, and any real constants *λ* ∈ (*γ*, 1), and $\gamma \in \left({0,1} \right)$, $\forall n \left({\in {\varvec{Z}}_{0+}} \right) \ge N$ since $$\left\{{T_{n}} \right\}\begin{array}{*{20}c} {_{\to}} \\ {^{\to}} \\ \end{array} \left\{{T} \right\}$$ a.s.. The following cases can occur in (5):

- $$F_{{x_{n}^{*},x^{*}}} \left({k^{-1} \eta t} \right) \ge F_{{x_{n}^{*},x^{*}}} (t) > 1 - \lambda;\forall n \left({\in {\varvec{Z}}_{0+}} \right) \ge N;\forall t \in {\varvec{R}}_{+}$$
  Then, {*x*_n_^*^} → x^*^ a.s. and $$\eta \in \left[{k,1} \right)$$ since $$F \in \varDelta_{\varvec{F}}$$ is non-decreasing and left-continuous.
- $$F_{{x_{{n_{j}}}^{*},x^{*}}} \left({k^{-1} \eta t} \right) \le min \left({F_{{x_{{n_{j}}}^{*},x^{*}}} (t),1 - \lambda} \right); \forall j \in {\varvec{Z}}_{0+}$$
  for a given $$\eta \in \left({0,k} \right]$$, some $$t \in {\varvec{R}}_{+}$$ and some subsequence $$\left\{{x_{{n_{j}}}^{*}} \right\} \subseteq \left\{{x_{n}^{*}} \right\}$$ of fixed points of $$\left\{{T_{{n_{j}}}} \right\} \subseteq \left\{{T_{n}} \right\}$$. If $$F_{{x_{{n_{j}}}^{*},x^{*}}} (t) > 1 - \lambda$$, one also finds that $$\left\{{x_{{n_{j}}}^{*}} \right\} \to x^{*}$$ a.s. with $$\eta \in \left({0,k} \right].$$ The convergence of the subsequence ensures that {*x*_n_^*^} → x^*^a.s. If $$F_{{x_{{n_{j}}}^{*},x^{*}}} (t) \le 1 - \lambda$$ and then $$F_{{x_{{n_{j}}}^{*},x^{*}}} \left({k^{-1} t_{1}} \right) \le 1 - \lambda$$ with $$t_{1} = k^{-1} t$$ and we deduce from (5) under the same reasoning with the replacement *t* → *t*_1_ that either $$F_{{x_{{n_{j}}}^{*},x^{*}}} \left({k^{-1} t_{1}} \right) > 1 - \lambda;\forall t_{1} \in {\varvec{R}}_{+}$$ and {*x*_n_^*^} → x^*^a.s. or $$F_{{x_{{n_{j}}}^{*},x^{*}}} \left({k^{-1} \eta t_{1}} \right) = F_{{x_{{n_{j}}}^{*},x^{*}}} \left({k^{- 2} \eta t} \right) \le 1 - \lambda$$ for some subsequence $$\left\{{x_{{n_{j}}}^{*}} \right\} \subseteq \left\{{x_{n}^{*}} \right\}$$ of fixed points of $$\left\{{T_{{n_{j}}}} \right\} \subseteq \left\{{T_{n}} \right\}$$. But, since $$1 = lim_{j \to \infty} lim_{i \to \infty} F_{{x_{{n_{j}}}^{*},x^{*}}} \left({k^{- i} \eta t} \right) = lim_{j \to \infty} lim_{{t_{i} \to \infty}} F_{{x_{{n_{j}}}^{*},x^{*}}} \left({\eta t_{i}} \right) \le 1 - \lambda$$ would be a contradiction, some finite *t*_*l*_ = *k*^−ℓ^*t* exists such that $$F_{{x_{{n_{j}}}^{*},x^{*}}} \left({t_{\ell}} \right) = F_{{x_{{n_{j}}}^{*},x^{*}}} \left({k^{{- \left({\ell + 1} \right)}} t} \right) > 1 - \lambda;\forall n \left({\in {\varvec{Z}}_{0+}} \right) \ge N_{\ell},\forall t \in {\varvec{R}}_{+}$$ and some $$N_{\ell} = N_{\ell} \left({\lambda,t} \right)$$ for any subsequence $$\left\{{x_{{n_{j}}}^{*}} \right\} \subseteq \left\{{x_{n}^{*}} \right\}$$ implying that $$lim_{n \to \infty} F_{{x_{n}^{*},x^{*}}} (t) = lim_{n \to \infty} F_{{x_{n}^{*},x^{*}}} \left({0^{+}} \right) = 1$$ so that {*x*_n_^*^} → x^*^a.s. On the other hand, for any given real constant $$\gamma \in (0,1)$$ and $$t \in {\varvec{R}}_{+}$$ and some *N* = *N*(*λ*, *t*), one gets, since $$\left\{{x_{n}^{*}} \right\} \to x^{*}$$, a.s. that
  $$F_{{T^{m} x_{n}^{*},x^{*}}} (t) = F_{{T^{m} x_{n}^{*},T^{m} x^{*}}} (t) \ge F_{{x_{n}^{*},x^{*}}} \left({k^{- m} t} \right) \ge F_{{x_{n}^{*},x^{*}}} (t) > 1 - \lambda;\quad \forall n \left({\in {\varvec{Z}}_{0+}} \right) \ge N;\;\forall m \in {\varvec{Z}}_{0+}$$
  and
  $${\mathop{lim}_{{t \to 0^{+}}}} {\mathop{lim}_{n \to \infty}} {\mathop{lim}_{m \to \infty}} F_{{T^{m} x_{n}^{*},x^{*}}} (t) = {\mathop{lim}_{{t \to 0^{+}}}} {\mathop{lim}_{n \to \infty}} F_{{T^{\ell} x_{n}^{*},x^{*}}} (t) = 1;\quad \forall \ell \in {\varvec{Z}}_{0+}$$
  so that, furthermore, $$\left\{{T^{m} x_{n}^{*}} \right\} \to x^{*}$$ a.s.; $$\forall m \in {\varvec{Z}}_{0+}$$ and *T*^*m*^*x*_n_^*^ → x^*^ a.s. as $$n, m \to \infty$$. Property (i) has been proved. On the other hand, note that
  6 $$\begin{aligned} F_{{T^{m} x_{n}^{*},x^{*}}} (t) & \ge \varDelta_{M} \left({F_{{T^{m} x_{n}^{*},T^{m} x^{*}}} (t/2),F_{{T^{m} x^{*},x^{*}}} (t/2)} \right) \hfill \\ & \ge \varDelta_{M} \left({F_{{T^{m} x_{n}^{*},T^{m} x^{*}}} (t/2),F_{{x^{*},x^{*}}} (t/2)} \right) \hfill \\ & \ge \varDelta_{M} \left({F_{{T^{m} x_{n}^{*},T^{m} x^{*}}} (t/2),1} \right) \hfill \\ & \ge F_{{x_{n}^{*},x^{*}}} \left({k^{- m} t/2} \right);\quad \forall t \in {\varvec{R}}_{+};\; \forall m,n \in {\varvec{Z}}_{+} \hfill \\ \end{aligned}$$
  and
  $$\begin{aligned} \mathop {lim}\limits_{m \to \infty} \mathop {lim}\limits_{n \to \infty} F_{{T^{m} x_{n}^{*},x^{*}}} (t) & \ge \mathop {lim}\limits_{m \to \infty} \mathop {lim}\limits_{n \to \infty} F_{{x_{n}^{*},x^{*}}} (t) \\ & = \mathop {lim}\limits_{n \to \infty} F_{{x_{n}^{*},x^{*}}} \left({0^{+}} \right) = F\left({+ \infty^{-}} \right) = 1;\forall t \in {\varvec{R}}_{+} \\ \end{aligned}$$
  since it has been already proved that $$\left\{{x_{n}^{*}} \right\} \to x^{*}$$ a.s., where *F*(+∞^−^) denotes the left-limit of *F*(*t*) as *t* → +∞. On the other hand, for any finite $$m \in {\varvec{Z}}_{+}$$, one has from (6) that $$F_{{T^{m} x_{n}^{*},x^{*}}} (t) \ge F_{{x_{n}^{*},x^{*}}} \left({k^{- m} t/2} \right) = F_{{x_{n}^{*},x^{*}}} \left({t_{1}} \right)$$ for $$t_{1} = t_{1} \left({t,m} \right) = k^{- m} t/2$$. Since $$t \in {\varvec{R}}_{+}$$, there exists $$N_{1} = N_{1} \left({\gamma, m,t} \right) \ge N$$ such that $$F_{{T^{m} x_{n}^{*},x^{*}}} (t) > 1 - \gamma;\forall n \left({\in {\varvec{Z}}_{0+}} \right) \ge N_{1}$$, where $$N = N\left({\gamma,t} \right) = min \left({z \in {\varvec{Z}}_{0+} : 
F_{{x_{n}^{*},x^{*}}} (t) > 1 - \gamma} \right)$$ for any given $$t \in {\varvec{R}}_{+}$$ and $$\gamma \in \left({0,1} \right)$$. Then,
  $$F_{T^{m} x_{n}^{*}, x^{*}} (t) \ge F_{x_{n}^{*},x^{*}} (t) \ge F_{x_{n}^{*}, x^{*}} (t_{1}) > 1 - \gamma\quad{\rm for}\;t \ge 2k^{m} t_{1}$$

Property (i) includes the convergence results {*x*_n_^*^} → x^*^a.s. and *T*^*m*^*x*_n_^*^ → x^*^a.s. as *n*, *m* → ∞. It is now proved that *T*^*m*^*x*_n_^*^ → x^*^ a.s. as *n* → ∞, $\forall m \in {\varvec{Z}}_{0+}$. Proceed by contradiction by assuming that $\left\{{T^{m} x_{n}^{*}} \right\} \neg \to x^{*}$ a.s. for some $m \in {\varvec{Z}}_{+}$ then there exists some real constant *γ*_1_ ∈ (0, 1)and some subsequence $\left\{{x_{{n_{j}}}^{*}} \right\} \subseteq \left\{{x_{n}^{*}} \right\}$ such that the following contradiction follows to $\left\{{x_{n}^{*}} \right\} \to x^{*}$ a.s.

$$1 - \gamma_{1} \ge \mathop {lim\;sup}\limits_{j \to \infty} \mathop {lim}\limits_{{t \to 0^{+}}} F_{{x_{{n_{j}}}^{*},x*}} (t) \ge \mathop {lim}\limits_{j \to \infty} \mathop {lim}\limits_{{t \to 0^{+}}} F_{{x_{{n_{j}}}^{*},x^{*}}} \left({k^{- m} t/2} \right) = 1$$

Then,

$$\mathop{lim}\limits_{n \to \infty} F_{{T^{m} x_{n}^{*},x^{*}}} (t) = \mathop{lim}\limits_{n \to \infty} F_{{T^{\ell} x_{n}^{*},x^{*}}} \left({0^{+}} \right) = \mathop{lim}\limits_{n,m \to \infty} F_{{T^{m} x_{n}^{*},x^{*}}} \left({0^{+}} \right) = 1;\quad \forall \ell \in {\varvec{Z}}_{0+} {\rm and} \left\{{T^{m} x_{n}^{*}} \right\} \to x^{*} {\rm a.s.};$$

$\forall m \in {\varvec{Z}}_{0+}$. Property (ii) has been proved.$\square$

The following auxiliary result is then used:

### **Lemma 2**

*The following properties hold:*

(i) *If *$T_{n}: X \to X,\forall n \in {\varvec{Z}}_{+}$* are all continuous and *$$\left\{T_{n}^{j}\right\}\begin{array}{*{20}c} {_{\to}} \\ {^{\to}} \\ \end{array} \left\{{T^{j}} \right\}$$* a.s., *$\forall j \in \bar{m}$* for some given *$m \in {\varvec{Z}}_{+}$*, where *$T: X \to X$*, then *$$\left\{{T_{n}^{m + 1}} \right\}\begin{array}{*{20}c} {_{\to}} \\ {^{\to}} \\ \end{array} \left\{{T^{m + 1}} \right\}$$* a.s.*
(ii) *If the self-maps *$T_{n} :X \to X,\forall n \in {\varvec{Z}}_{+}$* are continuous and *$$\left\{{T_{n}} \right\}\begin{array}{*{20}c} {_{\to}} \\ {^{\to}} \\ \end{array} \left\{{T} \right\}$$* a.s. then *$$\left\{{T_{n}^{m}} \right\}\begin{array}{*{20}c} {_{\to}} \\ {^{\to}} \\ \end{array} \left\{{T^{m}} \right\}$$* a.s., *$\forall m \in {\varvec{Z}}_{+}$.
(iii) *If the self-maps *$T_{n} :X \to X,\forall n \in {\varvec{Z}}_{+}$* are strictly contractive and *$$\left\{{T_{n}} \right\}\begin{array}{*{20}c} {_{\to}} \\ {^{\to}} \\ \end{array} \left\{{T} \right\}$$* a.s. then *$$\left\{{T_{n}^{m}} \right\}\begin{array}{*{20}c} {_{\to}} \\ {^{\to}} \\ \end{array} \left\{{T^{m}} \right\}$$*a.s.,*$\forall m \in {\varvec{Z}}_{+}$.
(iv) *If *$$\left\{{T_{n}} \right\}\begin{array}{*{20}c} {_{\to}} \\ {^{\to}} \\ \end{array} \left\{{T} \right\}$$* a.s. and T*_*n*_* commutes with T in X*, $\forall n \in {\varvec{Z}}_{0+}$* then *$$\left\{{T_{n}^{m}} \right\}\begin{array}{*{20}c} {_{\to}} \\ {^{\to}} \\ \end{array} \left\{{T^{m}} \right\}$$* a.s., *$\forall m \in {\varvec{Z}}_{+}$.

### *Proof*

Note that $T_{n}^{j+1}x = T_{n} \left(T_{n}^{j}x\right),\forall n \in {\varvec{Z}}_{+}$ and, since $$\left\{{T_{n}^{j}} \right\}\begin{array}{*{20}c} {_{\to}} \\ {^{\to}} \\ \end{array} \left\{{T^{j}} \right\}$$ a.s., $\forall j \in \bar{m}$, then *T*_*n*_^*j*^(*T*_ℓ_*x*) → *T*^*j*^(*T*_ℓ_*x*) as *n* → ∞; $\forall j \in \bar{m}$, $\forall \ell \in \bar{n}$, ∀*x* ∈ *X* and *T*^*j*^(*T*_*n*_*x*) → *T*^*j*^(*Tx*) = *T*^*j*+1^*x* as *n* → ∞ a.s., $\forall j \in \bar{m}$, ∀*x* ∈ *X* since $T_{n} :X \to X$ is continuous for any $n \in {\varvec{Z}}_{+}$, that is,

7 $$\begin{aligned} F_{{T_{n}^{j} \left({T_{n} x} \right),T^{j} \left({Tx} \right)}} (t) & \ge \varDelta_{M} \left({F_{{T_{n}^{j} \left({T_{n} x} \right),T^{j} \left({T_{n} x} \right)}} (t/2),F_{{T^{j} \left({T_{n} x} \right),T^{j} \left({Tx} \right)}} (t/2)} \right) \hfill \\ & \ge min \left({1 - \gamma,F_{{T^{j} \left({T_{n} x} \right),T^{j} \left({Tx} \right)}} (t/2)} \right) \hfill \\ \end{aligned}$$

for any given $t \in {\varvec{R}}_{+}$, *γ* ∈ (0, 1) and $\lambda \in \left({\gamma,1} \right),\forall j \in \overline{m - 1} \cup \{0\}$ and all $n \left({\in {\varvec{Z}}_{0+}} \right) \ge N_{1}$ and some $N_{1} \left({\in {\varvec{Z}}_{0+}} \right) = N_{1} \left({\gamma,t} \right)$ such that $max_{0 \le j \le m} F_{{T_{n}^{j} \left({T_{n} x} \right),T^{j} \left({T_{n} x} \right)}} (t/2) \ge 1 - \gamma > 1 - \lambda$ since $$\left\{{T_{n}^{j}} \right\}\begin{array}{*{20}c} {_{\to}} \\ {^{\to}} \\ \end{array} \left\{{T^{j}} \right\}$$ a.s., $\forall j \in \overline{m - 1} \cup \{0\}$. Since $T_{n} : X \to X$ is continuous $\forall n \in {\varvec{Z}}_{0+}$ there is $N_{2} \left({\in {\varvec{Z}}_{0+}} \right) = N_{2} \left({\lambda, t} \right)$ such that $F_{{T^{j} \left({T_{n} x} \right), T^{j} \left({Tx} \right)}} (t/2) > 1 - \lambda$ for all $n \left({\in {\varvec{Z}}_{0+}} \right) \ge N_{1}$, $\forall j \in {\varvec{Z}}_{0+}$, $\forall t \in {\varvec{R}}_{+}$ and any given real constant $\lambda \in (\gamma,1)$. Then, from (7),

8 $$\begin{aligned} &\mathop {min}\limits_{0 \le j \le m} F_{{T_{n}^{j + 1} x, T^{j + 1} x}} (t) \hfill \\ &\quad > min \left({1 - \lambda, 1 - \lambda} \right) = 1 - \lambda;\forall j \in \overline{m - 1},\forall t \in {\varvec{R}}_{+},\quad \forall n\left({\in {\varvec{Z}}_{0+}} \right) \ge max \left({N_{1}, N_{2}} \right) \hfill \\ \end{aligned}$$

Then,

9 $$\mathop{min}\limits_{0 \le j \le m + 1} F_{{T_{n}^{j} x, T^{j} x}} (t) > 1 - \lambda;\quad \forall t \in {\varvec{R}}_{+},\forall n\left({\in {\varvec{Z}}_{0+}} \right) \ge max \left({N_{1}, N_{2}} \right)$$

and ${min}_{0 \le j \le m + 1} lim_{n \to \infty} lim_{{t \to 0^{+}}} F_{{T_{n}^{j} x, T^{j} x}} (t) = 1$. Thus, *T*_*n*_^*j*^(*T*_*n*_*x*) → *T*^*j*+1^*x* a.s. as *n* → ∞, $\forall j \in \bar{m}$. Then, $$\left\{{T_{n}^{m + 1}} \right\}\begin{array}{*{20}c} {_{\to}} \\ {^{\to}} \\ \end{array} \left\{{T^{m + 1}} \right\}$$. Property (i) has been proved. Also, if $T_{n} :X \to X$, $\forall n \in {\varvec{Z}}_{+}$ are all continuous and $$\left\{{T_{n}} \right\}\begin{array}{*{20}c} {_{\to}} \\ {^{\to}} \\ \end{array} \left\{{T} \right\}$$ a.s., assumed it also holds that $$\left\{{T_{n}^{m}} \right\}\begin{array}{*{20}c} {_{\to}} \\ {^{\to}} \\ \end{array} \left\{{T^{m}} \right\}$$ a.s. for some $m \in {\varvec{Z}}_{+}$ then $$\left\{{T_{n}^{m + 1}} \right\}\begin{array}{*{20}c} {_{\to}} \\ {^{\to}} \\ \end{array} \left\{{T^{m + 1}} \right\}$$ a.s. via complete induction from Property (i) and Property (ii) follows as well. Property (iii) follows from Property (ii) since if the self-maps $T_{n} :X \to X$, $\forall n \in {\varvec{Z}}_{+}$ are continuous since they are strictly contractive.

On the other hand, if *T*_*n*_ commutes with *T* in *X* for all $n \in {\varvec{Z}}_{0+}$ then *T*^*j*−*i*^*T*_*n*_*T*^*i*^*x* = *T*_*n*_*T*^*j*^*x* = *T*^*j*^*T*_*n*_*x*; $\forall i\left({\le j} \right), j \in {\varvec{Z}}_{0+}$, $\forall n \in {\varvec{Z}}_{0+}$, ∀*x* ∈ *X* which used in (7) yields by using $$\left\{{T_{n}} \right\}\begin{array}{*{20}c} {_{\to}} \\ {^{\to}} \\ \end{array} \left\{{T} \right\}$$ a.s., $\forall n \in {\varvec{Z}}_{0+}$ and *T*_*n*_*Tx* = *TT*_*n*_*x*:

10 $$\begin{aligned} & \mathop {min}\limits_{0 \le j \le m} F_{{T_{n}^{j + 1} x, T^{j + 1} x}} (t) \hfill \\ & \quad \ge min \left({1 - \gamma, F_{{T_{n} \left({T^{j} x} \right), T\left({T^{j} x} \right)}} (t/2)} \right) = 1 - \lambda;\\ & \qquad \forall j \in \overline{m - 1},\forall t \in {\varvec{R}}_{+},\forall n\left({\in {\varvec{Z}}_{0+}} \right) \ge max \left({N_{1}, N_{2}} \right) \hfill \\ \end{aligned}$$

and any given $m \in {\varvec{Z}}_{0+}$. Then, $$\left\{{T_{n}} \right\}\begin{array}{*{20}c} {_{\to}} \\ {^{\to}} \\ \end{array} \left\{{T} \right\}$$ a.s. and Property (iv) is proved.$\square$

The subsequent result is concerned with the probabilistic convergence properties of the sequences $\left\{{x_{n}} \right\} \subset X$ generated by the iterated scheme $x_{n + 1} = T_{n} x_{n}$; $\forall n \in {\varvec{Z}}_{+}$ for any given *x*_1_ ∈ *X*:

### **Theorem 2**

*Let *$\left({X, {\varvec{F}}, \varDelta_{M}} \right)$* be a complete Menger space and let *$\{T_{n}\}$* be a sequence of operators *$T_{n} :X \to X$*, such that *$F_{{T_{n}}} = \left\{{x_{n}^{*}} \right\}$, $\forall n \in {\varvec{Z}}_{+}$, $$\left\{{T_{n}} \right\}\begin{array}{*{20}c} {_{\to}} \\ {^{\to}} \\ \end{array} \left\{{T} \right\}$$* a.s. and *$T: X \to X$*is a (strict) k-contraction with F*_*T*_ = {x^*^}.* Consider the sequence *$\left\{{x_{n}} \right\} \subset X$* generated by the iterated scheme *$x_{n + 1} = T_{n} x_{n}$; $\forall n \in {\varvec{Z}}_{+}$* for any given x*_1_ ∈ *X*.* Then, the following properties hold:*

(i) $T^{m} x_{n} \to x^{*}$* a.s. as m* → ∞; $\forall n \in {\varvec{Z}}_{0+}$* and *$T^{m} x_{n} \to x^{*}$* a.s. as *$n, m \to \infty$,
(ii) $$\begin{aligned} & F_{{T^{m} x_{n + 1}, T^{m} x_{n + 1}^{0}}} (t) \ge F_{{T_{n} x_{n}, Tx_{n}}} \left({k^{- m} t} \right); \quad \forall n \in {\varvec{Z}}_{0+},\; \forall t \in {\varvec{R}}_{+}, \; \forall m \in {\varvec{Z}}_{0+}\\ & F_{{T^{m} x_{n + 1}, T^{m} x_{n + 1}^{0}}} (t) \ge F_{{T_{n} x_{n}, Tx_{n}}} \left({k^{- m} t} \right) > 1 - \gamma; \forall n \left({\in {\varvec{Z}}_{0+}} \right) \ge N_{1}, \quad \forall t \in {\varvec{R}}_{+}, \; \forall m \in {\varvec{Z}}_{0+} \\ & \mathop{lim}\limits_{m \to \infty} F_{{T^{m} x_{n + 1}, T^{m} x_{n + 1}^{0}}} (t) = 1; \quad \forall n \left({\in {\varvec{Z}}_{0+}} \right) \ge N_{1},\; \forall t \in {\varvec{R}}_{+} \\ & \mathop{lim}\limits_{m, n \to \infty} F_{{T^{m} x_{n + 1}, T^{m} x_{n + 1}^{0}}} (t) = 1;\quad \forall t \in {\varvec{R}}_{+}, \end{aligned}$$
  *wherex*_*n*+1_^0^ = *Tx*_*n*_, *for some*$N_{1} \left({\in {\varvec{Z}}_{0+}} \right) = N_{1} \left({\gamma, t, m} \right),$
(iii) *Assume that some of the conditions below holds:*
  1. $$\left\{{T_{n}^{m}} \right\} \begin{array}{*{20}c} {_{\to}} \\ {^{\to}} \\ \end{array} T^{m}$$* a.s.*; $$\forall m \in {\varvec{Z}}_{+}$$
  2. $$\{T_{n}\} \begin{array}{*{20}c} {_{\to}} \\ {^{\to}} \\ \end{array} T$$* a.s. and either *$T_{n} : X \to X$* is continuous for all*$n \in {\varvec{Z}}_{0+}$* or T*_*n*_* commutes with T for all *$n \in {\varvec{Z}}_{0+}$.
    *Then, *$$\left\{{x_{n}} \right\} \to x^{*}$$* a.s.*
(iv) *If *$\left({X, {\varvec{F}}, \varDelta_{M}} \right)$* is a (non-necessarily complete) Menger space, each elements of the sequence *$\{T_{n}\}$* of operators *$T_{n} :X \to X$* has a unique fixed points *$F_{{T_{n}}} = \left\{{x_{n}^{*}} \right\}, \forall n \in {\varvec{Z}}_{+},$* and commute everywhere in X with a (strict) k-contraction *$T: X \to X$* of unique fixed point F*_*T*_ = {x^*^}, *then:*
  $$\mathop{lim\;inf}\limits_{n \to \infty} F_{{x_{n}, x^{*}}} (t) \ge \mathop{lim\;sup}\limits_{j,(n - j) \to \infty} F_{x_{n},({T_{n}. \ldots. T_{n - j + 1}. T_{n - j}}) x^{*}} \left({t^{-}} \right);\quad \forall t \in {\varvec{R}}_{+}$$

### *Proof*

For any given $\gamma \in (0,1)$ and $t \in {\varvec{R}}_{+}$, there exists $N_{1} \left({\in {\varvec{Z}}_{0+}} \right) = N_{1} \left({\gamma, t} \right)$ such that $F_{{T_{n} x_{n}, Tx_{n}}} (t) > 1 - \gamma$ since $$\left\{{T_{n}} \right\}\begin{array}{*{20}c} {_{\to}} \\ {^{\to}} \\ \end{array} \left\{{T} \right\}$$ a.s.. On the other hand, since $T: X \to X$ be a (strict) *k*-contraction with a unique fixed point x^*^ ∈ *X*, one gets for any given any given $\gamma \in (0,1)$ and some $N_{1} \left({\in {\varvec{Z}}_{0+}} \right) = N \left({\gamma, k^{- m} t} \right)$ that:

$$F_{{T^{m} x_{n}, x^{*}}} (t) = F_{{T^{m} x_{n}, T^{m} x^{*}}} (t) \ge F_{{x_{n}, x^{*}}} \left({k^{- m} t} \right)$$

and then $T^{m} x_{n} \to x^{*}$ a.s. as *m* → ∞; $\forall n \in {\varvec{Z}}_{0+}$ and $T^{m} x_{n} \to x^{*}$ a.s. as *n*, *m* → ∞. On the other hand, if *x*_*n*+1_ = *T*_*n*_*x*_*n*_; $\forall n \in {\varvec{Z}}_{0+}$ for any *x*_1_ ∈ *X*, since $$\left\{ {T_{n}} \right\}\begin{array}{*{20}c} {_{\to}} \\ {^{\to}} \\ \end{array} \left\{{T} \right\}$$ a.s. and $T:X \to X$ is *k*-contractive, then

$$\begin{aligned} & F_{{T^{m} x_{n + 1}, T^{m} \left({Tx_{n}} \right)}} (t) = F_{{T^{m} T_{n} x_{n}, T^{m + 1} x_{n}}} (t) \ge F_{{T_{n} x_{n}, Tx_{n}}} \left({k^{- m} t} \right) > 1 - \gamma; \quad \forall n \left({\in {\varvec{Z}}_{0+}} \right) \ge N_{1}, ; \forall t \in {\varvec{R}}_{+}, \; \forall m \in {\varvec{Z}}_{0+} \\ & \mathop {lim}\limits_{m \to \infty} F_{{T^{m} x_{n + 1}, T^{m + 1} x_{n}}} (t) = \mathop {lim}\limits_{m \to \infty} F_{{T^{m} T_{n} x_{n}, T^{m + 1} x_{n}}} (t) = 1; \quad \forall n \left({\in {\varvec{Z}}_{0+}} \right) \ge N_{1}, \quad \forall t \in {\varvec{R}}_{+}\\ & \mathop {lim}\limits_{m, n \to \infty} F_{{T^{m} T_{n} x_{n}, T^{m + 1} x_{n}}} (t) = 1;\quad \forall t \in {\varvec{R}}_{+}\end{aligned}$$

Properties (i) and (ii) have been proved.

To prove Property (iii), proceed by contradiction by assuming that $\left\{{x_{n}} \right\} \neg \to x^{*}$ a.s.. Then, there exists some real constant $\gamma_{0} \in (0,1)$ such that the following contradiction holds for some subsequence $\left\{{T_{n}^{m}} \right\}$ of $\{T_{n}\}$ which generates the sequence $x_{{n_{j} + 1}} = T_{{n_{j}}} x_{{n_{j}}}$ for some given *x*_1_ ∈ *X*, $\forall j \in {\varvec{Z}}_{0+}$:

11 $$\begin{aligned} 1 - \gamma_{0} & \ge \mathop {lim\;sup}\limits_{j \to \infty} \left({\mathop {lim\;sup}\limits_{m \to \infty} F_{{x_{{n_{j} + m}}, x^{*}}} (t)} \right) = \mathop {lim\;sup}\limits_{j \to \infty} \left({\mathop {lim\;sup}\limits_{m \to \infty} F_{{T_{{n_{j}}}^{m} x_{{n_{j}}}, x^{*}}} (t)} \right) \\ & \ge \mathop {lim\;sup}\limits_{j \to \infty} \left({\mathop {lim\;sup}\limits_{m \to \infty} \varDelta_{M} \left({F_{{T_{{n_{j}}}^{m} x_{{n_{j}}}, T^{m} x_{{n_{j}}}}} (t/2), F_{{T^{m} x_{{n_{j}}}, T^{m} x^{*}}} (t/2)} \right)} \right) \\ & \ge \varDelta_{M} \left({\mathop{lim\; inf}\limits_{j \to \infty} \left({\mathop {lim\;sup}\limits_{m \to \infty} F_{{T_{{n_{j}}}^{m} x_{{n_{j}}}, T^{m} x_{{n_{j}}}}} (t/2)} \right), \mathop {lim}\limits_{m \to \infty} F_{{x_{{n_{j}}}, x^{*}}} \left({k^{- m} t/2} \right)} \right) \\ & = min (1,1) = 1 \\ \end{aligned}$$

from Lemma 2, since either $$\left\{{T_{{n_{j}}}^{m}} \right\} \begin{array}{*{20}c} {_{\to}} \\ {^{\to}} \\ \end{array} T^{m}$$ a.s., $\forall m \in {\varvec{Z}}_{+}$, or $$\{T_{n}\} \begin{array}{*{20}c} {_{\to}} \\ {^{\to}} \\ \end{array} T$$ a.s. with $T_{n} : X \to X$ being either everywhere continuous or it commutes with $T : X \to X$, $\forall n \in {\varvec{Z}}_{0+}$. Note that (7) yields the contradiction *γ*_0_ ≤ 0. Thus, $\left\{{x_{n}} \right\} \neg \to x^{*}$ a.s. is false and $\left\{{x_{{n_{j}}}} \right\} \to x^{*}$ a.s. and then $\left\{{x_{n}} \right\} \to x^{*}$ a.s. Property (iii) has been proved.

To prove Property (iv), note that for any real constant $\sigma \in (0,1)$:

12 $$\begin{aligned} F_{{x_{n + m}, x^{*}}} (t) & = F_{{\bar{T} \left({n, n + m} \right) x_{n}, x^{*}}} (t) \\ & \ge \varDelta_{M} \left({F_{{\bar{T} \left({n, n + m} \right) x_{n}, T^{m} x_{n + m}}} \left({\sigma t} \right), F_{{T^{m} x_{n + m}, T^{m} x^{*}}} \left({\left({1 - \sigma} \right) t} \right)} \right) \\ & \ge \varDelta_{M} \left({F_{{\bar{T} \left({n, n + m} \right) x_{n}, \bar{T} \left({n, n + m} \right) T^{m} x_{n}}} \left({\sigma t} \right), F_{{x_{n + m}, x^{*}}} \left({k^{- m} \left({1 - \sigma} \right) t} \right)} \right) \\ & \ge F_{{\bar{T} \left({n, n + m} \right) x_{n}, \bar{T} \left({n, n + m} \right) T^{m} x_{n}}} \left({\sigma t} \right) \\ & \ge \varDelta_{M} \left({F_{{\bar{T} \left({n, n + m} \right) x_{n}, \bar{T} \left({n, n + m} \right) T^{m} x^{*}}} \left({\sigma^{2} t} \right), F_{{\bar{T} \left({n, n + m} \right) T^{m} x^{*}, \bar{T} \left({n, n + m} \right) T^{m} x_{n}}} \left({\sigma \left({1 - \sigma} \right) t} \right)} \right) \\ & = \varDelta_{M} \left({F_{{\bar{T} \left({n, n + m} \right) x_{n}, \bar{T} \left({n, n + m} \right) T^{m} x^{*}}} \left({\sigma^{2} t} \right), F_{{T^{m} \bar{T} \left({n, n + m} \right) x^{*}, T^{m} \bar{T} \left({n, n + m} \right) x_{n}}} \left({\sigma \left({1 - \sigma} \right) t} \right)} \right) \\ & \ge \varDelta_{M} \left({F_{{x_{n + m}, \bar{T} \left({n, n + m} \right) T^{m} x^{*}}} \left({\sigma^{2} t} \right), F_{{\bar{T} \left({n, n + m} \right)x^{*}, x_{n + m}}} \left({k^{- m} \sigma \left({1 - \sigma} \right) t} \right)} \right) ;\\ \end{aligned}$$

$\forall j \in {\varvec{Z}}_{0+}, \; \forall t \in {\varvec{R}}_{+}$, since the elements of $\{T_{n}\}$ commute with *T*, $F_{{x_{n + m}, x^{*}}} \left({k^{- m} \sigma \left({1 - \sigma} \right)} \right) \ge F_{{x_{n + m}, x^{*}}} (t)$, $\forall n, m \in {\varvec{Z}}_{+}$ and $lim_{m \to \infty} F_{{\bar{T} \left({n, n + m} \right)x^{*}, x_{n + m}}} \left({k^{- m} \sigma \left({1 - \sigma} \right)} \right) = 1$, $\forall t \in {\varvec{R}}_{+}$,where $\bar{T} (n, n + m) = T_{n + m - 1}\cdot \cdots \cdot T_{n}$ is a composite operator with m consecutive members of the sequence {*T*_*n*_}. Thus, for any given $t \in {\varvec{R}}_{+}$ and $\lambda \in (0,1)$, there exist $m_{0}, n_{0} \in {\varvec{Z}}_{0+}$ such that for any $n \left({\in {\varvec{Z}}_{0+}} \right) \ge n_{0}$ and $m \left({\in {\varvec{Z}}_{0+}} \right) \ge m_{0}$, $F_{{\bar{T} \left({n, n + m} \right)x^{*}, x_{n + m}}} \left({k^{- m} t/4} \right) > 1 - \lambda$. Then, one gets from (12), since x^*^ = *T*^*m*^x^*^ that

$$F_{{x_{n + m}, x^{*}}} (t) \ge F_{{x_{n + m}, \bar{T} \left({n, n + m} \right) x^{*}}} \left({\sigma^{2} t} \right);\quad\forall n \ge n_{0},\; \forall m \ge m_{0},\forall t \in {\varvec{R}}_{+}$$

$$\mathop{lim \; inf}\limits_{n \to \infty} F_{{x_{n}, x^{*}}} (t) \ge \mathop {lim\;sup}\limits_{n \to \infty} F_{{x_{n}, \bar{T} \left({n - m, n} \right) x^{*}}} \left({\sigma^{2} t} \right);\quad \forall m \ge m_{0},\;\forall t \in {\varvec{R}}_{+}$$

for any $\sigma \in (0,1)$ and renaming subscripts, the above inequality becomes identical to:

13 $$F_{{x_{n}, x^{*}}} (t) \ge F_{{x_{n}, \bar{T} \left({j, n} \right) x^{*}}} \left({\sigma^{2} t} \right);\quad \forall j\left({\in {\varvec{Z}}_{0+}} \right) \ge n_{0},m\left({\in {\varvec{Z}}_{0+}} \right) \ge m_{0},\;\forall t \in {\varvec{R}}_{+}$$

14 $$\begin{aligned}\mathop{lim \; inf}\limits_{n \to \infty} F_{{x_{n}, x^{*}}} (t) & \ge \mathop {lim}\limits_{{\sigma \to 1^{-}}} \left({\mathop {lim\;sup}\limits_{n \to \infty} F_{{x_{n}, \bar{T} \left({n - m, n} \right) x^{*}}} \left({\sigma^{2} t} \right)} \right) \\ & = \mathop {lim\;sup}\limits_{n \to \infty} F_{{x_{n}, \bar{T} \left({n - m, n} \right) x^{*}}} \left({t^{-}} \right);\quad m\left({\in {\varvec{Z}}_{0+}} \right) \ge m_{0},\; \forall t \in {\varvec{R}}_{+} \end{aligned}$$

and Property (iv) is proved.$\square$

## Main results concerning the point-wise convergence of operators

The first result of this section is the following one:

### **Theorem 3**

*Let *$\left({X, {\varvec{F}}, \varDelta_{M}} \right)$* be a complete Menger space and let *$\{T_{n}\}$* be a sequence of operators such that:*

1. *The operators *$T_{n} :X \to X$; $\forall n \in {\varvec{Z}}_{0+}$* of the sequence* {*T*_*n*_} *are all strict k-contractions for some real constant*$k \in (0,1)$,
2. $$\{T_{n}\} \to T$$* for some *$$T :X \to X$$.

Then, $T: X \to X$ is a strict *k*-contraction and $\left\{{x_{n}^{*}} \right\} \to x^{*}$ a.s., where $F_{{T_{n}}} = \left\{{x_{n}^{*}} \right\}$; $\forall n \in {\varvec{Z}}_{+}$, and *F*_*T*_ = {x^*^}. Furthermore, $\left\{{x_{n}} \right\} \to x^{*}$ a.s., where *x*_*n*+1_ = *T*_*n*_*x*_*n*_; $\forall n \in {\varvec{Z}}_{0+}$ for any given *x*_0_ ∈ *X*.

### *Proof*

We have that $F_{{T_{n} x, T_{n} y}} (t) \ge F_{x,y} \left({k^{-1} t} \right)$; $\forall n \in {\varvec{Z}}_{0+},\forall t \in {\varvec{R}}_{+}$ for any $x, y \in X$, and

15 $$\begin{aligned} F_{Tx,Ty} (t) & \ge \varDelta_{M} \left({F_{{Tx, T_{n} x}} (t/2), F_{{T_{n} x, Ty}} (t/2)} \right) \hfill \\ & \ge \varDelta_{M} \left({F_{{Tx, T_{n} x}} (t/2), \varDelta_{M} \left({F_{{T_{n} x, T_{n} y}} (t/4), F_{{T_{n} y, Ty}} (t/4)} \right)} \right) \hfill \\ & \ge \varDelta_{M} \left({F_{{Tx, T_{n} x}} (t/2), \varDelta_{M} \left({F_{x,y} \left({k^{-1} t/4} \right), F_{{T_{n} y, Ty}} (t/4)} \right)} \right);\quad \forall n \in {\varvec{Z}}_{0+},\; \forall t \in {\varvec{R}}_{+} \hfill \\ \end{aligned}$$

Thus, since $\{T_{n}\} \to T$, $T_{n} : X \to X$ are strict *k*-contractions, then everywhere continuous, and $\varDelta_{M} : [0,1] \times [0,1] \to [0,1]$ is a continuous triangular norm,

16 $$\begin{aligned} F_{Tx,Ty} (t) & \ge \varDelta_{M} \left({\mathop{lim \; inf}\limits_{n \to \infty} F_{{Tx, T_{n} x}} (t/2), \varDelta_{M} \left({F_{x,y} \left({k^{-1} t/4} \right), \mathop{lim \; inf}\limits_{n \to \infty} F_{{T_{n} y, Ty}} (t/4)} \right)} \right) \hfill \\ &\ge \varDelta_{M} \left({\mathop{lim \; inf}\limits_{n \to \infty} F_{{Tx, T_{n} x}} (t/2), \varDelta_{M} \left({F_{x,y} \left({k^{-1} t/4} \right), \mathop{lim \; inf}\limits_{n \to \infty} F_{{T_{n} y, Ty}} (t/4)} \right)} \right) \hfill \\ &= \varDelta_{M} \left({1, \varDelta_{M} \left({F_{x,y} \left({k^{-1} t/4} \right), 1} \right)} \right) \hfill \\ &= \varDelta_{M} \left({F_{x,y} \left({k^{-1} t/4} \right), 1} \right) \hfill \\ &= F_{x,y} \left({k^{-1} t/4} \right);\quad \forall n \in {\varvec{Z}}_{0+},\;\forall t \in {\varvec{R}}_{+},\;\forall x, y \in X \hfill \\ \end{aligned}$$

so that

17 $$F_{{T^{n} x, T^{n} y}} (t) \ge F_{x,y} \left({k^{- n} t/4} \right);\forall n \in {\varvec{Z}}_{0+},\quad \forall t \in {\varvec{R}}_{+},\; \forall x, y \in X$$

18 $$\mathop {lim}\limits_{n \to \infty} F_{{T^{n} x, T^{n} y}} (t) = 1;\quad \forall t \in {\varvec{R}}_{+},\; \forall x, y \in X$$

Eq. (17), leading to (18), establishes that $T: X \to X$ is a strict *k*-contraction. It has to be proved that it has a unique fixed point x^*^. Assume on the contrary that there are two $x^{*}, \bar{x}^{*} \left({\ne x^{*}} \right) \in F(T)$ so that

$$F_{{\bar{x}^{*}, x^{*}}} (t) = F_{{T^{n} \bar{x}^{*}, T^{n} x^{*}}} (t) \ge F_{{\bar{x}^{*}, x^{*}}} \left({k^{- n} t} \right);\quad \forall n \in {\varvec{Z}}_{0+},\; \forall t \in {\varvec{R}}_{+}$$

$$F_{{\bar{x}^{*}, x^{*}}} (t) = \mathop {lim}\limits_{n \to \infty} F_{{T^{n} \bar{x}^{*}, T^{n} x^{*}}} (t) \ge \mathop {lim}\limits_{n \to \infty} F_{{\bar{x}^{*}, x^{*}}} \left({k^{- n} t} \right) = 1;\quad \forall t \in {\varvec{R}}_{+}$$

and then $\bar{x}^{*} = x^{*}$ by the property 1 of (1) of the PM space $\left({X,{\varvec{F}}} \right)$.On the other hand, one gets by taking *y* = *y*(*x*) = *Tx* and *z*_*n*_ = *z*_*n*_(*x*) = *T*^*n*^*x* for any given *x* ∈ *X* that

$$\mathop {lim}\limits_{n \to \infty} F_{{T^{n + 1} x, T^{n} x}} (t) = \mathop {lim}\limits_{n \to \infty} F_{{Tz_{n}, z_{n}}} (t) = 1;\quad \forall t \in {\varvec{R}}_{+},\; \forall x \in X$$

and, for any given $\lambda \in (0,1)$ and $t \in {\varvec{R}}_{+}$, there is $N_{0} = N_{0} \left({\varepsilon, \lambda} \right) \in {\varvec{Z}}_{0+}$ such that $F_{{z_{n + 1}, z_{n}}} (t) > 1 - \lambda; \forall n\left({\in {\varvec{Z}}_{0+}} \right) \ge N_{0}, \forall t \in {\varvec{R}}_{+}$ so that $\left\{{z_{n}} \right\}$ is a Cauchy sequence which converges to some limit point z^*^ = z^*^(*x*) ∈ *X* since $\left({X, {\varvec{F}}, \varDelta_{M}} \right)$ is complete. Since the fixed point x^*^of $T: X \to X$ is unique, any limit point z^*^ = z^*^(*x*) ∈ *X* of any sequence $\left\{{T^{n} x} \right\}$ for any arbitrary initial point *x* ∈ *X* is the fixed point x^*^ of $T: X \to X$. It remains to prove that $\left\{{x_{n}} \right\} \to x^{*}$ a.s., where *x*_*n*+1_ = *T*_*n*_*x*_*n*_;$\forall n \in {\varvec{Z}}_{0+}$, for any given initial point *x*_0_ ∈ *X*. Note that

19 $$\begin{aligned} & F_{{x_{n + 1}, x^{*}}} (t) \\ & \quad \ge \varDelta_{M} \left({F_{{x_{n + 1}, T_{n + 1}^{m} x_{n + 1}}} (t/2), \varDelta_{M} \left({F_{{T_{n + 1}^{m} x_{n + 1}, T^{m} x_{n + 1}^{*}}} (t/4), F_{{T^{m} x_{n + 1}^{*}, T^{m} x^{*}}} (t/4)} \right)} \right) \\ &\quad \ge \varDelta_{M} \left({F_{{x_{n + 1}, T_{n + 1}^{m} x_{n + 1}}} (t/2), \varDelta_{M} \left({\varDelta_{M} \left({F_{{T_{n + 1}^{m} x_{n + 1}, T_{n + 1}^{m} x_{n + 1}^{*}}} (t/8), F_{{T^{m} x_{n + 1}^{*},T_{n + 1}^{m} x_{n + 1}^{*}}} (t/8)} \right), F_{{T^{m} x_{n + 1}^{*}, T^{m} x^{*}}} (t/4)} \right)} \right) \\ &\quad \ge \varDelta_{M} \left({F_{{x_{n + 1}, T_{n + 1}^{m} x_{n + 1}}} (t/2), \varDelta_{M} \left({\varDelta_{M} \left({F_{{x_{n + 1}, x_{n + 1}^{*}}} \left({k^{- m} t/8} \right), F_{{T^{m} x_{n + 1}^{*},T_{n + 1}^{m} x_{n + 1}^{*}}} (t/8)} \right), F_{{x_{n + 1}^{*}, x^{*}}} \left({k^{- m} t/4} \right)} \right)} \right); \\ & \qquad \forall n \in {\varvec{Z}}_{0+},\; \forall m \in {\varvec{Z}}_{+},\; \forall t \in {\varvec{R}}_{+} \\ \end{aligned}$$

Note also that

20 $$\begin{aligned}F_{{T^{m} x_{n + 1}^{*},T_{n + 1}^{m} x_{n + 1}^{*}}} (t) & = F_{{T^{p} \left({T^{m - p} x_{n + 1}^{*}} \right), T_{n}^{p} \left({T_{n + 1}^{m - p} x_{n + 1}^{*}} \right)}} (t) \\ & = F_{{T\left({T^{m - 1} x_{n + 1}^{*}} \right), T_{n} \left({T_{n + 1}^{m - 1} x_{n + 1}^{*}} \right)}} (t) \end{aligned}$$

for any $p,n, \left({m \ge p} \right) \in {\varvec{Z}}_{0+}, \forall t \in {\varvec{R}}_{+}$ and, since $T_{n} :X \to X; \forall n \in {\varvec{Z}}_{0+}$ and $T :X \to X$ are all strict *k*-contractions, they are also continuous so that if $\{T_{n}\} \to T$, then $\left\{{T_{n} x} \right\} \to Tx$ a.s. and $lim_{n \to \infty} F_{{T_{n} x, Tx}} (t) = 1; \forall t \in {\varvec{R}}_{+}$ as *n* → ∞ for any given *x* ∈ *X*. Then, $\left\{{T_{n}^{p} \left({T_{n + 1}^{m - p} x_{n + 1}^{*}} \right)} \right\} \to T^{p} \left({T^{m - p} x_{n + 1}^{*}} \right)$ a.s. as *m* → ∞, $\forall n \in {\varvec{Z}}_{0+}$ since $T_{n} :X \to X; \forall n \in {\varvec{Z}}_{0+}$ and $T :X \to X$ are strict *k*-contractions. This implies from (20) that

21 $$\mathop {lim}\limits_{m \to \infty} F_{{T^{m} x_{n + 1}^{*}, T_{n + 1}^{m} x_{n + 1}^{*}}} (t) = 1;\quad \forall t \in {\varvec{R}}_{+},\; \forall n \in {\varvec{Z}}_{0+}$$

On the other hand,

22 $$\left\{{T_{n}^{m} x_{n}} \right\} \to x_{n}^{*} \; {\rm a.s.\;as\;}m \to \infty;\quad \forall n \in {\varvec{Z}}_{0+}$$

since $\{T_{n}\}$ is a sequence of strict *k*-contractive operators with $F_{{T_{n}}} = \left\{{x_{n}^{*}} \right\}; \forall n \in {\varvec{Z}}_{0+}$. Furthermore, one has:

23 $$\mathop {lim}\limits_{m \to \infty} F_{{x_{n + 1}, x_{n + 1}^{*}}} \left({k^{- m} t} \right) = \mathop {lim}\limits_{m \to \infty} F_{{x_{n + 1}^{*}, x^{*}}} \left({k^{- m} t/4} \right) = 1;\quad \forall t \in {\varvec{R}}_{+}$$

In a similar way to (19), we get:

24 $$\begin{aligned} F_{{x_{n + 1}^{*}, x^{*}}} (t) & = F_{{T_{n + 1}^{m} x_{n + 1}^{*}, T^{m} x^{*}}} (t) \\ & \ge \varDelta_{M} \left({F_{{T^{m} x_{n + 1}^{*}, x^{*}}} (t/2), F_{{T^{m} x_{n + 1}^{*}, T_{n + 1}^{m} x_{n + 1}^{*}}} (t/2)} \right) \hfill \\ & \ge \varDelta_{M} \left({F_{{x_{n + 1}^{*}, x^{*}}} \left({k^{- m} t/2} \right), F_{{T^{m} x_{n + 1}^{*}, T_{n + 1}^{m} x_{n + 1}^{*}}} (t/2)} \right);\quad \forall m \in {\varvec{Z}}_{0+}, \; \forall t \in {\varvec{R}}_{+} \hfill \\ \end{aligned}$$

and taking limits as *m* → ∞, one gets that $lim_{n \to \infty} F_{{T^{m} x_{n + 1}^{*}, T_{n + 1}^{m} x_{n + 1}^{*}}} (t/2) = lim_{n \to \infty} F_{{x_{n + 1}^{*}, x^{*}}} (t) = 1; \forall t \in {\varvec{R}}_{+}$ so that $\left\{{x_{n}^{*}} \right\} \to x^{*}$ a.s.. On the other hand, the use of (21)–(23) in (19) as well as $lim_{n \to \infty} F_{{x_{n + 1}^{*}, x^{*}}} (t) = 1; \forall t \in {\varvec{R}}_{+}$ got from (24) yields for all $t \in {\varvec{R}}_{+}$, since $\varDelta_{M} : [0,1] \times [0,1] \to [0,1]$ is a continuous triangular norm,

25 $$\begin{aligned} & \mathop{lim\;inf}\limits_{n \to \infty} F_{{x_{n + 1}, x^{*}}} (t) \hfill \\ & \quad \ge \mathop{lim\;inf}\limits_{n, m \to \infty} \varDelta_{M} \left({F_{{x_{n + 1}, T_{n + 1}^{m} x_{n + 1}}} (t/2), \varDelta_{M} \left({\varDelta_{M} \left({F_{{x_{n + 1}, x_{n + 1}^{*}}} \left({k^{- m} t/8} \right), F_{{T^{m} x_{n + 1}^{*},T_{n + 1}^{m} x_{n + 1}^{*}}} (t/8)} \right), F_{{x_{n + 1}^{*}, x^{*}}} \left({k^{- m} t/4} \right)} \right)} \right) \hfill \\ &\quad \ge \varDelta_{M} \left({\mathop {lim}\limits_{n, m \to \infty} F_{{x_{n + 1}, T_{n + 1}^{m} x_{n + 1}}} (t/2), \varDelta_{M} \left({\varDelta_{M} \left({\mathop {lim}\limits_{m \to \infty} F_{{x_{n + 1}, x_{n + 1}^{*}}} \left({k^{- m} t/8} \right), \mathop {lim}\limits_{m \to \infty} F_{{T^{m} x_{n + 1}^{*},T_{n + 1}^{m} x_{n + 1}^{*}}} (t/8)} \right), \mathop {lim}\limits_{m \to \infty} F_{{x_{n + 1}^{*}, x^{*}}} \left({k^{- m} t/4} \right)} \right)} \right) \hfill \\ &\quad \ge \varDelta_{M} \left({1, \varDelta_{M} \left({\varDelta_{M} \left({1, 1} \right), 1} \right)} \right) = 1 \hfill \\ \end{aligned}$$

so that $\exists lim_{n \to \infty} F_{{x_{n}, x^{*}}} (t) = 1$; $\forall t \in {\varvec{R}}_{+}$ and $\left\{{x_{n}} \right\} \to x^{*}$ a.s..$\square$

As it occurs in the deterministic counterpart, (Berinde 2007), the uniform convergence of a sequence of operators $\{T_{n}\}$ can be weakened if such operators possess certain additional contractive properties. See also Istratescu (1981). In this case, it is possible to get some close properties to those proved in “Main results concerning the uniform convergence of operators” section for the case of uniform convergence. Firstly, two definitions which are then used follow below:

### **Definition 2**

(Berinde 2007) A non-decreasing function $\varphi : {\varvec{R}}_{0+} \to {\varvec{R}}_{0+}$ (i.e. $\varphi$(*t*_1_) ≤ $\varphi$(*t*_2_) if *t*_1_ ≤ *t*_2_) is said to be a comparison function if $\left\{{\varphi^{n} (t)} \right\} \to 0, \forall t \in {\varvec{R}}_{+}$. If, furthermore, $\left({t - \varphi (t)} \right) \to + \infty$ as *t* → + ∞ then it is said to be a strict comparison function.

### *Example 2*

Note that $\varphi (t) = \frac{\lambda (t) t}{1 + \lambda (t) t}$ for $t \in {\varvec{R}}_{0+}$ with $\lambda : {\varvec{R}}_{0+} \to {\varvec{R}}_{0+}$ being such that $\lambda (t) t$ is non-decreasing is a strict comparison function since it is non-decreasing and $\varphi^{n} (t) = \varphi \left({\varphi^{n - 1} (t)} \right) = \frac{\lambda (t) t}{1 + n \lambda (t) t}$ for all $n \in {\varvec{Z}}_{+}$ implying that $\left\{{\varphi^{n} (t)} \right\} \to 0; \forall t \in {\varvec{R}}_{0+}$ as *n* → ∞.

### *Example 3*

Let $\left({X,{\varvec{F}}} \right)$ be a PM-space, let $T: X \to X$ be a mapping on *X* and let $\varphi :X \times X \times {\varvec{R}}_{0+} \to {\varvec{R}}_{0+}$ be defined as $\varphi_{x,y} (t) = \frac{\lambda (t) \left(F_{x,y}^{- 1} (t) - 1\right)}{1 + \lambda (t) \left(F_{x,y}^{-1} (t) - 1\right)}; \forall x, y \in X, \forall t \in {\varvec{R}}_{0+}$ leading to the *n*-the composite function with itself resulting to be $\varphi_{x,y}^{n} (t) = \frac{\lambda (t) \left(F_{x,y}^{-1} (t) - 1\right)}{1 + n\lambda (t) \left(F_{x,y}^{-1} (t) -1\right)}; \forall t \in {\varvec{R}}_{0+}, \forall n \in {\varvec{Z}}$ which satisfies $\left\{{\varphi^{n} (t)} \right\} \to 0; \forall t \in {\varvec{R}}_{0+}$ as *n* → ∞. Then, $\varphi :X \times X \times {\varvec{R}}_{0+} \to {\varvec{R}}_{0+}$ is a strict comparison function for any $x, y \in X$ provided that *λ*(0) = 0 and $\lambda (t) \left({F_{x,y}^{-1} (t) - 1} \right)$ is non-decreasing for all $t \in {\varvec{R}}_{0+}$ for each pair $\left({x,y} \right) \in X \times X$. Note, in particular, that $\varphi$_*x*,*x*_(*t*) = 0; $\forall t \in {\varvec{R}}_{0+}$; ∀*x* ∈ *X* so that the null-function $\varphi$ is both non-increasing and non-decreasing.

### **Definition 3**

Let $\left({X,{\varvec{F}}} \right)$ be a PM-space. Then, $G : X \to X$ is said to be a strict $\varphi$-contraction if $G_{Tx,Ty}^{-1} (t) \le 1 + \varphi \left({G_{x,y}^{-1} (t) - 1} \right)$, ∀*x*, *y* ∈ *X*,$\forall t \in {\varvec{R}}_{+}$ for some strict comparison function $\varphi :{\varvec{R}}_{0+} \to {\varvec{R}}_{0+}$.

The next result follows:

### **Theorem 4**

*Let *$\left({X, {\varvec{F}}, \varDelta_{M}} \right)$* be a complete Menger space and let *$\{T_{n}\}$* be a sequence of operators such that:*

1. *The operators *$T_{n} :X \to X$* of the sequence* {*T*_*n*_} *are all strict*$\varphi$*-contractions*,
2. $$\{T_{n}\} \to T$$* for some *$$T :X \to X$$.

Then, $T: X \to X$ is a strict $\varphi$-contraction and $\left\{{x_{n}^{*}} \right\} \to x^{*}$ a.s., where $F_{{T_{n}}} = \left\{{x_{n}^{*}} \right\}; \forall n \in {\varvec{Z}}_{+}$ and *F*_*T*_ = {x^*^}. Furthermore $\left\{{x_{n}} \right\} \to x^{*}$ a.s., where *x*_*n*+1_ = *T*_*n*_*x*_*n*_; $\forall n \in {\varvec{Z}}_{0+}$ for any given *x*_0_ ∈ *X*.

### *Proof*

We have $F_{{T_{n} x,T_{n} y}}^{-1} (t) - 1 \le \varphi \left({F_{x,y}^{-1} (t) - 1} \right)$; ∀*x*, *y* ∈ *X*,$\forall t \in {\varvec{R}}_{+}, \forall n \in {\varvec{Z}}_{0+}$ for some strict comparison function $\varphi :{\varvec{R}}_{0+} \to {\varvec{R}}_{0+}$, since all the operators of the sequence {*T*} are strict _*n*_$\varphi$-contractions, what is equivalent to

$$F_{T_{n} x, T_{n} y} (t) \ge \frac{1}{1 + \varphi \left(F_{x,y}^{- 1} (t) - 1\right)};\quad \forall x, y \in X,\; \forall t \in {\varvec{R}}_{+}, \; \forall n \in {\varvec{Z}}_{0+}$$

and then

26 $$\begin{aligned} F_{Tx,Ty} (t) & \ge \varDelta_{M} \left({F_{{Tx, T_{n} x}} (t/2), F_{{T_{n} x, Ty}} (t/2)} \right) \hfill \\ &\ge \varDelta_{M} \left({F_{{Tx, T_{n} x}} (t/2), \varDelta_{M} \left({F_{{T_{n} x, T_{n} y}} (t/4), F_{{T_{n} y, Ty}} (t/4)} \right)} \right) \hfill \\ &\ge \varDelta_{M} \left({F_{{Tx, T_{n} x}} (t/2), \varDelta_{M} \left({\frac{1}{{1 + \varphi \left({F_{x,y}^{-1} (t/4) - 1} \right)}}, F_{{T_{n} y, Ty}} (t/4)} \right)} \right);\\ & \quad \forall n \in {\varvec{Z}}_{0+},\;\forall t \in {\varvec{R}}_{+} \hfill \\ \end{aligned}$$

Thus, since $\{T_{n}\} \to T$, $T_{n} : X \to X$ all strict $\varphi$-contractions, then everywhere continuous, and $\varDelta_{M} : [0,1] \times [0,1] \to [0,1]$ is a continuous triangular norm, one gets:

27 $$\begin{aligned} F_{Tx,Ty} (t) &\ge \mathop{lim\;inf}\limits_{n \to \infty} \varDelta_{M} \left({F_{{Tx, T_{n} x}} (t/2), \varDelta_{M} \left({\frac{1}{{1 + \varphi \left({F_{x,y}^{-1} (t/4) - 1} \right)}}, F_{{T_{n} y, Ty}} (t/4)} \right)} \right) \hfill \\ &\ge \varDelta_{M} \left({\mathop{lim\;inf}\limits_{n \to \infty} F_{{Tx, T_{n} x}} (t/2), \varDelta_{M} \left({\frac{1}{{1 + \varphi \left({F_{x,y}^{-1} (t/4) - 1} \right)}}, \mathop{lim\;inf}\limits_{n \to \infty} F_{{T_{n} y, Ty}} (t/4)} \right)} \right) \hfill \\ &= \varDelta_{M} \left({1, \varDelta_{M} \left({\frac{1}{{1 + \varphi \left({F_{x,y}^{-1} (t/4) - 1} \right)}}, 1} \right)} \right) \hfill \\ &= \varDelta_{M} \left({\frac{1}{{1 + \varphi \left({F_{x,y}^{-1} (t/4) - 1} \right)}}, 1} \right) \hfill \\ &= \frac{1}{{1 + \varphi \left({F_{x,y}^{-1} (t/4) - 1} \right)}};\quad \forall t \in {\varvec{R}}_{+},\; \forall x, y \in X \hfill \\ \end{aligned}$$

so that

28 $$\begin{aligned} F_{Tx, Ty}^{-1} (t) & \le 1 + \varphi \left(F_{x, y}^{-1} (t/4) -1 \right) \\ F_{T^{n}x, T^{n} y}^{-1} (t) & \le 1 + \varphi \left(F_{T^{n - 1} x, T^{n - 1}y}^{- 1} (t/4) - 1 \right) \le \cdots \le 1 + \varphi^{n} \left(F_{x, y}^{- 1} \left(t/2^{n + 1} \right) - 1 \right); \\ & \qquad \forall n \in {\varvec{Z}}_{0+}, \; \forall t \in {\varvec{R}}_{+},\; \forall x, y \in X\end{aligned}$$

since $lim_{n \to \infty} \varphi^{n} \left({F_{x,y}^{-1} \left({t/2^{n + 1}} \right) - 1} \right) = 0$ since $\left\{{\varphi^{n} (t)} \right\} \to 0; \forall t \in {\varvec{R}}_{+}$. Then, $lim_{n \to \infty} F_{x,y}^{-1} \left({t/2^{n + 1}} \right) = 1, \forall t \in {\varvec{R}}_{+}$, ∀*x*, *y* ∈ *X*. Thus, one also has that:

29 $$\mathop {lim}\limits_{n \to \infty} F_{{T^{n} x, T^{n} y}} (t) = 1;\quad \forall t \in {\varvec{R}}_{+},\; \forall x, y \in X$$

Eq. (28), leading to (29), establishes that $T: X \to X$ is a strict $\varphi$-contraction. It has to be proved that it has a unique fixed pointx^*^. Assume on the contrary that there are two points $x^{*}, \bar{x}^{*} \left({\ne x^{*}} \right) \in F(T)$ so that

30 $$F_{{\bar{x}^{*}, x^{*}}} (t) = F_{{T^{n} \bar{x}^{*}, T^{n} x^{*}}} (t) = \mathop {lim}\limits_{n \to \infty} F_{{T^{n} \bar{x}^{*}, T^{n} x^{*}}} (t) \le 1 + \mathop {lim}\limits_{n \to \infty} \varphi^{n} \left({F_{x,y}^{-1} \left({t/2^{n + 1}} \right) - 1} \right) = 1;\quad \forall t \in {\varvec{R}}_{+}$$

and then $\bar{x}^{*} = x^{*}$ by the property 1 of (1) of the PM space $\left({X,{\varvec{F}}} \right)$. On the other hand, one gets from (29) by taking *y* = *y*(*x*) = *Tx* and *z*_*n*_ = *z*_*n*_(*x*) = *T*^*n*^*x* for any given *x* ∈ *X* that

$$\mathop {lim}\limits_{n \to \infty} F_{{T^{n + 1} x, T^{n} x}} (t) = \mathop {lim}\limits_{n \to \infty} F_{{Tz_{n}, z_{n}}} (t) = 1;\quad \forall t \in {\varvec{R}}_{+},\; \forall x \in X$$

and, for any given $\lambda \in (0,1)$ and $t \in {\varvec{R}}_{+}$, there is $N_{0} = N_{0} \left({\varepsilon, \lambda} \right) \in {\varvec{Z}}_{0+}$ such that $F_{{z_{n + 1}, z_{n}}} (t) > 1 - \lambda; \forall n\left({\in {\varvec{Z}}_{0+}} \right) \ge N_{0}, \forall t \in {\varvec{R}}_{+}$ so that $\left\{{z_{n}} \right\}$ is a Cauchy sequence which converges to some limit point z^*^ = z^*^(*x*) ∈ *X* since $\left({X, {\varvec{F}}, \varDelta_{M}} \right)$ is complete. Since the fixed point x^*^of $T: X \to X$ is unique, any limit point z^*^ = z^*^(*x*) ∈ *X* of any sequence $\left\{{T^{n} x} \right\}$ for any arbitrary initial point *x* ∈ *X* is the fixed point x^*^ of $T: X \to X$. It remains to prove that $\left\{{x_{n}} \right\} \to x^{*}$ a.s., where *x*_*n*+1_ = *T*_*n*_*x*_*n*_; $\forall n \in {\varvec{Z}}_{0+}$, for any given initial point *x*_0_ ∈ *X*. Note that, since *T*_*n*_ is a strict $\varphi$-contraction with $F_{{T_{n}}} = \left\{{x_{n}^{*}} \right\}; \forall n \in {\varvec{Z}}_{0+}$, we can perform the two next replacements to a close set of inequalities to those got in (19) and (24) within the proof of Theorem 1

$$F_{{T_{n + 1}^{m} x_{n + 1}, T_{n + 1}^{m} x_{n + 1}^{*}}} (t) \to \frac{1}{{1 + \varphi^{m} \left({F_{{x_{n + 1}, x_{n + 1}^{*}}}^{-1} (t) - 1} \right)}}$$

$$F_{{x_{n + 1}^{*}, x^{*}}} \left({k^{- m} t} \right) \to \frac{1}{{1 + \varphi^{m} \left({F_{{x_{n + 1}^{*}, x^{*}}}^{-1} (t) - 1} \right)}}$$

for all $t \in {\varvec{R}}_{+}$ and $m \in {\varvec{Z}}_{0+}$. Thus, one gets instead of (24),

31 $$\begin{aligned} F_{{x_{n + 1}^{*}, x^{*}}} (t) & = F_{{T_{n + 1}^{m} x_{n + 1}^{*}, T^{m} x^{*}}} (t) \ge \varDelta_{M} \left({F_{{T^{m} x_{n + 1}^{*}, x^{*}}} (t/2), F_{{T^{m} x_{n + 1}^{*}, T_{n + 1}^{m} x_{n + 1}^{*}}} (t/2)} \right) \hfill \\ &\ge \varDelta_{M} \left({\frac{1}{{1 + \varphi^{m} \left({F_{{x_{n + 1}^{*}, x^{*}}}^{-1} (t/2) - 1} \right)}}, F_{{T^{m} x_{n + 1}^{*}, T_{n + 1}^{m} x_{n + 1}^{*}}} (t/2)} \right);\quad \forall n,m \in {\varvec{Z}}_{0+},\; \forall t \in {\varvec{R}}_{+} \hfill \\ \end{aligned}$$

Since $\left\{{T_{n}^{m}} \right\} \to T^{m}$ then $lim_{n \to \infty} F_{{T^{m} x_{n + 1}^{*}, T_{n + 1}^{m} x_{n + 1}^{*}}} (t/2) = 1; \forall t \in {\varvec{R}}_{+}; \forall m \in {\varvec{Z}}_{0+}, \forall t \in {\varvec{R}}_{+}$ and the above constraint implies that

$$\mathop{lim\;inf}\limits_{n \to \infty} \left({\frac{1}{{F_{{x_{n + 1}^{*}, x^{*}}}^{-1} (t)}} - \frac{1}{{1 + \varphi^{m} \left({F_{{x_{n + 1}^{*}, x^{*}}}^{-1} (t/2) - 1} \right)}}} \right) \ge 0;\quad \forall m \in {\varvec{Z}}_{0+},\;\forall t \in {\varvec{R}}_{+}$$

Equivalently, ${lim\;sup}_{n \to \infty} \left({F_{{x_{n + 1}^{*}, x^{*}}}^{-1} (t) - 1 - \varphi \left({F_{{x_{n + 1}^{*}, x^{*}}}^{-1} (t/2) - 1} \right)} \right) \le 0;\forall t \in {\varvec{R}}_{+}$. Since, furthermore, *F*^−1^(*t*) is non-increasing and $\varphi$(*t*) is non-decreasing, one has:

$$\begin{aligned} &\mathop {lim\;sup}\limits_{n \to \infty} \left({F_{{x_{n + 1}^{*}, x^{*}}}^{-1} (t) - 1 - \varphi \left({F_{{x_{n + 1}^{*}, x^{*}}}^{-1} (t) - 1} \right)} \right) \hfill \\ &\quad \le \mathop{lim\;sup}\limits_{n \to \infty} \left({F_{{x_{n + 1}^{*}, x^{*}}}^{-1} (t) - 1 - \varphi \left({F_{{x_{n + 1}^{*}, x^{*}}}^{-1} (t/2) - 1} \right)} \right) \le 0;\quad \forall t \in {\varvec{R}}_{+}; \hfill \\ \end{aligned}$$

Define $\sigma_{\eta} = sup \left\{{\sigma \in {\varvec{R}}_{0+} :\sigma - \varphi \left(\sigma \right) \le \eta} \right\}$ which is defined for any given $\eta \in {\varvec{\bar{R}}}_{0+}$ (the extended nonnegative real semi-line) from the property of strict $\varphi$-contractions, since $\varphi$ is a strict comparison function, ${lim\;sup}_{t \to + \infty} \left({t - \varphi^{m} (t)} \right) \le lim_{t \to + \infty} \left({t - \varphi (t)} \right) = + \infty; \forall n \in {\varvec{Z}}_{0+}$ as *t* → + ∞. It turns out that $lim_{\eta \to 0} \sigma_{\eta} = 0$. Now, define $\sigma_{n} = F_{{x_{n}^{*}, x^{*}}}^{-1} (t) - 1; \forall n \in {\varvec{Z}}_{0+}, \forall t \in {\varvec{R}}_{+}$. Taking into account that

32 $$\mathop {lim\;sup}\limits_{n \to \infty} \left({F_{{x_{n}^{*}, x^{*}}}^{-1} (t) - 1 - \varphi \left({F_{{x_{n}^{*}, x^{*}}}^{-1} (t) - 1} \right)} \right) \le 0;\quad \forall t \in {\varvec{R}}_{+}$$

One concludes that $lim_{\eta \to 0} \sigma_{\eta} = lim_{\eta \to 0} \left({F_{{x_{n}^{*}, x^{*}}}^{-1} (t) - 1} \right) = 0; \forall t \in {\varvec{R}}_{+}$ so that {*x*_n_^*^} → x^*^ a.s..

Also, one can get, instead of (19) in the proof of Theorem 1, that

$$\begin{aligned} F_{{x_{n + 1}, x^{*}}} (t) & \ge \varDelta_{M} \left({F_{{x_{n + 1}, T_{n + 1}^{m} x_{n + 1}}} (t/2), \varDelta_{M} \left({F_{{T_{n + 1}^{m} x_{n + 1}, T^{m} x_{n + 1}^{*}}} (t/4), F_{{T^{m} x_{n + 1}^{*}, T^{m} x^{*}}} (t/4)} \right)} \right) \\ & \ge \varDelta_{M} \left({F_{{x_{n + 1}, T_{n + 1}^{m} x_{n + 1}}} (t/2), \varDelta_{M} \left({\varDelta_{M} \left({F_{{T^{m} x_{n + 1}^{*},T_{n + 1}^{m} x_{n + 1}^{*}}} (t/8), \frac{1}{{1 + \varphi^{m} \left({F_{{x_{n + 1}, x_{n + 1}^{*}}}^{-1} (t/8) - 1} \right)}}} \right), \frac{1}{{1 + \varphi^{m} \left({F_{{x_{n + 1}^{*}, x^{*}}}^{-1} (t/4) - 1} \right)}}} \right)} \right); \forall n \in {\varvec{Z}}_{0+},\forall m \in {\varvec{Z}}_{+},\; \forall t \in {\varvec{R}}_{+} \\ \end{aligned}$$

and then $$lim_{n \to \infty} \varphi^{n} \left({F_{x,y}^{-1} \left({t/2^{n + 1}} \right) - 1} \right) = 0$$ since $$\left\{{\varphi^{n} (t)} \right\} \to 0$$; $$\forall t \in {\varvec{R}}_{+}$$. Take limits in the above expression by using the continuity of the minimum triangular norm and the fact that $$\varphi$$ is a strict $$\varphi$$-comparison function by using close arguments to those used to get (32). We then conclude in a similar way the validity of (21) to (25) by replacing the conditions of *k*-contractions by conditions of strict $$\varphi$$-contractions so that there exist the limits $$lim_{n \to \infty} F_{{x_{n}^{*}, x^{*}}} (t)$$$$= lim_{n \to \infty} F_{{x_{n}, x^{*}}} (t) = 1$$; $$\forall t \in {\varvec{R}}_{+}$$ and $$\left\{{x_{n}} \right\} \to x^{*}$$ a.s..$$\square$$

 A close result to Theorem 4 is now got in the case when $$\{T_{n}\} \begin{array}{*{20}c} {_{\to}} \\ {^{\to}} \\ \end{array} T$$, with $T_{n} : X \to X$, so that $T : X \to X$ is a strict $\varphi$-contraction without requesting that all the elements of the sequence {*T*_*n*_} be strict $\varphi$-contraction.

### **Theorem 5**

*Let *$\left({X, {\varvec{F}}, \varDelta_{M}} \right)$* be a complete Menger space and let *$\{T_{n}\}$* be a sequence of operators such that:*

1. $$\{T_{n}\} \begin{array}{*{20}c} {_{\to}} \\ {^{\to}} \\ \end{array} T$$* such that *$T_{n} :X \to X$; $\forall n \in {\varvec{Z}}_{0+}$* for some *$T :X \to X$* which is a strict *$\varphi$*-contraction,*
2. $$x_{n}^{*} \in F_{{T_{n}}} \ne \varnothing; \forall n \in {\varvec{Z}}_{0+}$$

Then, {*T*_*n*_} has a subsequence of strict $\varphi$-contractions and $\left\{{x_{n}^{*}} \right\} \to x^{*}$ a.s., where $F_{{T_{n}}} = \left\{{x_{n}^{*}} \right\}; \forall n \in {\varvec{Z}}_{+}$ and *F*_*T*_ = {x^*^}. Furthermore $\left\{{x_{n}} \right\} \to x^{*}$ a.s., where *x*_*n*+1_ = *T*_*n*_*x*_*n*_; $\forall n \in {\varvec{Z}}_{0+}$ for any given *x*_0_ ∈ *X*.

### *Proof*

We have $F_{Tx,Ty}^{-1} (t) - 1 \le \varphi \left({F_{x,y}^{-1} (t) - 1} \right)$; ∀*x*, *y* ∈ *X*,$\forall t \in {\varvec{R}}_{+}, \forall n \in {\varvec{Z}}_{0+}$ for some strict comparison function $\varphi :{\varvec{R}}_{0+} \to {\varvec{R}}_{0+}$, since $T: X \to X$ is a strict $\varphi$-contraction, equivalently, $F_{Tx,Ty} (t) \ge \frac{1}{{1 + \varphi \left({F_{x,y}^{-1} (t) - 1} \right)}}$; ∀*x*, *y* ∈ *X*,$\forall t \in {\varvec{R}}_{+}, \forall n \in {\varvec{Z}}_{0+}$, and then

$$\begin{aligned} F_{{T_{n} x, T_{n} y}} (t) & \ge \varDelta_{M} \left({F_{{T_{n} x, Tx}} (t/2), F_{{Tx, T_{n} y}} (t/2)} \right) \hfill \\ &\ge \varDelta_{M} \left({F_{{Tx, T_{n} x}} (t/2), \varDelta_{M} \left({F_{Tx,Ty} (t/4), F_{{T_{n} y, Ty}} (t/4)} \right)} \right) \hfill \\ &\ge \varDelta_{M} \left({F_{{Tx, T_{n} x}} (t/2), \varDelta_{M} \left({\frac{1}{{1 + \varphi \left({F_{x,y}^{-1} (t/4) - 1} \right)}}, F_{{T_{n} y, Ty}} (t/4)} \right)} \right);\quad \forall n \in {\varvec{Z}}_{0+},\; \forall t \in {\varvec{R}}_{+}. \hfill \\ \end{aligned}$$

Thus, since $$\{T_{n}\} \begin{array}{*{20}c} {_{\to}} \\ {^{\to}} \\ \end{array} T$$ and $T : X \to X$ is a strict $\varphi$-contraction, then everywhere continuous, and $\varDelta_{M} : [0,1] \times [0,1] \to [0,1]$ is a continuous triangular norm, one gets:

$$\begin{aligned} \mathop{lim\;inf}\limits_{n \to \infty} F_{{T_{n} x, T_{n} y}} (t) & \ge \mathop{lim\;inf}\limits_{n \to \infty} \varDelta_{M} \left({F_{{Tx, T_{n} x}} (t/2), \varDelta_{M} \left({\frac{1}{{1 + \varphi \left({F_{x,y}^{-1} (t/4) - 1} \right)}}, F_{{T_{n} y, Ty}} (t/4)} \right)} \right) \hfill \\ &\ge \varDelta_{M} \left({\mathop{lim\;inf}\limits_{n \to \infty} F_{{Tx, T_{n} x}} (t/2), \varDelta_{M} \left({\frac{1}{{1 + \varphi \left({F_{x,y}^{-1} (t/4) - 1} \right)}}, \mathop{lim\;inf}\limits_{n \to \infty} F_{{T_{n} y, Ty}} (t/4)} \right)} \right) \hfill \\ &= \varDelta_{M} \left({1, \varDelta_{M} \left({\frac{1}{{1 + \varphi \left({F_{x,y}^{-1} (t/4) - 1} \right)}}, 1} \right)} \right) = \frac{1}{{1 + \varphi \left({F_{x,y}^{-1} (t/4) - 1} \right)}};\quad \forall t \in {\varvec{R}}_{+},\; \forall x, y \in X \hfill \\ \end{aligned}$$

so that one gets the following recursion:

$$\begin{aligned} & \mathop{lim\;sup}\limits_{m \to \infty} \left(\mathop{lim\;sup}\limits_{n \to \infty} F_{T_{n}^{m}x, T_{n}^{m}y}^{- 1}(t) \right) \\ &\quad \le 1 + \mathop{lim\;sup}\limits_{m \to \infty} \left(\mathop{lim\;sup}\limits_{n \to \infty} \varphi \left(F_{{T_{n}}^{m - 1}x, T_{n}^{m - 1} y}^{- 1} (t/4) - 1 \right) \right) \\ &\quad \le \cdots \le 1 + \mathop{lim\;sup}\limits_{m \to \infty} \left(\mathop{lim\;sup}\limits_{n \to \infty} \varphi^{m} \left(F_{x, y}^{- 1} \left(t/2^{m + 1}\right) - 1 \right) \right) = 1 \\ \end{aligned}$$

since $lim_{m \to \infty} \varphi^{m} \left({F_{x,y}^{-1} \left({t/2^{n + 1}} \right) - 1} \right) = 0$ since $\left\{{\varphi^{m} (t)} \right\} \to 0; \forall t \in {\varvec{R}}_{+}$. Then, $lim_{m \to \infty} F_{x,y}^{-1} \left({t/2^{m + 1}} \right) = 1, \forall t \in {\varvec{R}}_{+}$, ∀*x*, *y* ∈ *X*, and $lim_{n \to \infty} F_{{T^{n} x, T^{n} y}} (t) = 1; \forall t \in {\varvec{R}}_{+}$, ∀*x*, *y* ∈ *X*. So, there is a subsequence $\left\{{T_{{n_{n}}}} \right\}$ of $\{T_{n}\}$ whose elements are strict $\varphi$-contractions and the elements of such a subsequence have unique fixed points $\left\{{x_{{n_{k}}}^{*}} \right\}$. Eq. (32) can also be got under the conditions of this theorem so that one concludes that {*x*_n_^*^} → x^*^ a.s.. The remaining of the proof is close to its counterpart in Theorem 4.$\square$

We now reformulate close results to the above ones associated with strict $\varphi$-contractions via a dual class of contractions referred to as dual strict $\varphi$-contractions which operate directly on contractive conditions on the probability density function instead on its inverse. For that purpose, we first introduce the concept of dual strict comparison function as follows:

### **Definition 4**

A non-increasing function $\varphi : {\varvec{R}}_{0+} \to {\varvec{R}}_{0+}$ (i.e. $\varphi$(*t*_1_) ≥ $\varphi$(*t*_2_) if *t*_1_ ≤ *t*_2_) is said to be a dual comparison function if $\left\{{\varphi^{n} (t)} \right\} \to 1, \forall t \in {\varvec{R}}_{+}$. If, furthermore, $\left({t - \varphi (t)} \right) \to + \infty$ as *t* → + ∞ then it is said to be a dual strict comparison function.

### **Definition 5**

Let (*X*, ***F***) be a PM-space. Then, $G : X \to X$ is said to be a dual strict $\varphi$-contraction if $G_{Tx,Ty} (t) \ge \varphi \left({G_{x,y} (t)} \right)$, ∀*x*, *y* ∈ *X*,$\forall t \in {\varvec{R}}_{+}$ for some dual strict comparison function $\varphi :{\varvec{R}}_{0+} \to {\varvec{R}}_{0+}$.

Note that if *T* is any strict *k*-contraction for any given $k \in (0,1)$ then

$$F_{Tx,Ty} (t) \ge F_{x,y} \left({k^{-1} t} \right) \ge F_{x,y} (t) = \varphi \left({F_{x,y} (t)} \right);\forall x,y \in X;\quad \forall t \in {\varvec{R}}_{+}$$

if $\varphi$ ≡ 1 which is a dual strict comparison function since *F* is non-decreasing and left-continuous.

If $\left\{{\varphi^{n} (t)} \right\} \to 1$, although $\varphi :{\varvec{R}}_{0+} \to {\varvec{R}}_{0+}$ be non-necessarily unity but a dual strict comparison function, then

$$F_{{T^{n + 1} x,T^{n + 1} y}} (t) \ge F_{Tx,Ty} \left({k^{- n} t} \right) \ge \varphi \left({F_{x,y} \left({k^{- n} t} \right)} \right) = \varphi^{n} \left({F_{x,y} (t)} \right);\quad\forall x,y \in X,\;\forall t \in {\varvec{R}}_{+}.$$

Since $\left\{{\varphi^{n} (t)} \right\} \to 1, \forall t \in {\varvec{R}}_{+}$ because it is a dual strict comparison function, all the limits of the above chain equalize unity. So, if $T:X \to X$ is any strict *k*-contraction then it is also a dual strict $\varphi$-contraction. The converse is not true in general. Assume now that $T:X \to X$ is a dual strict $\varphi$-contraction for some dual strict comparison function $\varphi$ so that $F_{Tx,Ty} (t) \ge \varphi \left({F_{x,y} (t)} \right); \forall x,y \in X, \forall t \in {\varvec{R}}_{+}$. Since *F* is non-decreasing and $\varphi$ is non-increasing, we have for $k \in (0,1)$:

$$F_{x,y} \left({k^{-1} t} \right) \ge F_{x,y} (t);\varphi \left({F_{x,y} \left({k^{-1} t} \right)} \right) \le \varphi \left({F_{x,y} (t)} \right);\quad \forall x,y \in X,\;\forall t \in {\varvec{R}}_{+}$$

so that $F_{Tx,Ty} (t) \ge \varphi \left({F_{x,y} (t)} \right) \ge \varphi \left({F_{x,y} \left({k^{-1} t} \right)} \right); \forall x,y \in X, \forall t \in {\varvec{R}}_{+}$ and any given *k* ∈ (0, 1). One then gets that $F_{{T^{n} x,T^{n} y}} (t) \ge \varphi^{n} \left({F_{x,y} \left({k^{- n} t} \right)} \right)$ so that $F_{{T^{n} x,T^{n} y}} (t) \to 1$ as *n* → ∞; ∀*x*, *y* ∈ *X*, $\forall t \in {\varvec{R}}_{+}$. Then, if $T:X \to X$ is a $\varphi$-contraction, it is not a strict *k*-contraction, in general.

The next result follows:

### **Theorem 6**

*Let *$\left({X, {\varvec{F}}, \varDelta_{M}} \right)$* be a complete Menger space and let *$\{T_{n}\}$* be a sequence of operators such that:*

1. *The operators *$T_{n} :X \to X; \forall n \in {\varvec{Z}}_{0+}$* of the sequence *{*T*_*n*_}* are all dual strict*$\varphi$*-contractions,*
2. $$\{T_{n}\} \to T$$* for some *$$T :X \to X$$.

Then, $T: X \to X$ is a dual strict $\varphi$-contraction and $\left\{{x_{n}^{*}} \right\} \to x^{*}$ a.s., where $F_{{T_{n}}} = \left\{{x_{n}^{*}} \right\}$; $\forall n \in {\varvec{Z}}_{+}$ and *F*_*T*_ = {x^*^}. Furthermore $\left\{{x_{n}} \right\} \to x^{*}$ a.s., where *x*_*n*+1_ = *T*_*n*_*x*_*n*_; $\forall n \in {\varvec{Z}}_{0+}$ for any given *x*_0_ ∈ *X*.

### *Proof*

We have $F_{{T_{n} x,T_{n} y}} (t) \ge \varphi \left({F_{x,y} (t)} \right)$; ∀*x*, *y* ∈ *X*,$\forall t \in {\varvec{R}}_{+}, \forall n \in {\varvec{Z}}_{0+}$ for some strict comparison function $\varphi :{\varvec{R}}_{0+} \to {\varvec{R}}_{0+}$, since all the operators of the sequence {*T*_*n*_} are dual strict $\varphi$-contractions; ∀*x*, *y* ∈ *X*,$\forall t \in {\varvec{R}}_{+}$. Under close steps to those used in the proof of Theorem 4, we get instead of (28):

33 $$F_{T^{n}x, T^{n}y} (t) \ge \varphi \left(F_{T^{n - 1} x, T^{n - 1}y} (t/4)\right) \ge \cdots \ge \varphi^{n} \left(F_{x, y} \left(t/2^{n + 1}\right)\right);\quad \forall n \in {\varvec{Z}}_{0+}, \; \forall t \in {\varvec{R}}_{+},\forall x, y \in X$$

and $lim_{n \to \infty} \varphi^{n} \left({F_{x,y} \left({t/2^{n + 1}} \right)} \right) = 1$, since $\left\{{\varphi^{n} (t)} \right\} \to 1; \forall t \in {\varvec{R}}_{+}$. Then $lim_{n \to \infty} F_{{T^{n} x, T^{n} y}} (t) = 1; \forall t \in {\varvec{R}}_{+}$, ∀*x*, *y* ∈ *X* and one can conclude in a similar way to Theorem 4 that *T*, which is the point-wise limit of the sequence $\{T_{n}\}$ of dual strict $\varphi$-contractions, is also a dual strict $\varphi$-contraction. We also can prove the remaining properties of the statement in a close way to the proof of Theorem 4.$\square$

It turns out that a similar result to Theorem 6, under the guidelines of Theorem 5, can be directly formulated for the case when $$\{T_{n}\} \begin{array}{*{20}c} {_{\to}} \\ {^{\to}} \\ \end{array} T$$ with $T_{n} :X \to X$ where the limit operator $T :X \to X$ is a dual strict $\varphi$-contraction.

## Numerical example

This section contains some numerical examples illustrating the theoretical results previously established, in particular Theorems 1, 3 and 6 from “Main results concerning the uniform convergence of operators” and “Main results concerning the point-wise convergence of operators” sections, respectively. For this purpose, we extend the example proposed in de la Sen and Ibeas (2015):

### *Example 4*

Consider the space $X = {\varvec{R}}_{0+} = \left[{0, + \infty} \right)$ with the probabilistic metric given by $F_{x,y} (t) = \frac{t}{t + d(x,y)}$ where *d*(*x*, *y*) is a deterministic metric, selected in this example as *d*(*x*, *y*) = ‖*x* − *y*‖ where ‖ · ‖ stands for the Euclidean norm. Clearly, $\left({\left[{0, + \infty} \right),F_{x,y} (t),\varDelta_{M}} \right)$ is a Menger PM-space. Moreover, consider now the iterative scheme given by *x*_*n*+1_ = *T*_*n*_*x*_*n*_; $n \in {\varvec{Z}}_{0+}$ with the family of nonlinear operators *T*_*n*_ (*n* ≥ 0) being defined by $T_{n} x_{n} = \frac{{x_{n}}}{{f(n)\left({1 + x_{n}} \right)}}$ on [0, + ∞) and $f(n) = \frac{2n + 3}{n + 1}$. This family of nonlinear operators is simultaneously a strict *k* and dual strict $\varphi$-contraction (according to Definitions 1 and 4, respectively) so that conditions from Theorems 1, 3 and 6 hold and they can be applied simultaneously. A numerical simulation of the iterative scheme is performed in this section to show these theoretical results. Initially, we must ensure that they are strict *k* and dual strict $\varphi$-contractions. Thus, according to Definition 1 each *T*_*n*_ has to satisfy $F_{{T_{n} x,T_{n} y}} (kt) \ge F_{x,y} (t)$, ∀*x*, *y* ∈ [0, + ∞) and ∀*t* ≥ 0 for some real constant 0 < *k* < 1. Therefore,

$$T_{n} x - T_{n} y = \frac{x}{f(n)(1 + x)} - \frac{y}{f(n)(1 + y)} = \frac{x - y}{f(n)(1 + x)(1 + y)}$$

so that

$$F_{{T_{n} x,T_{n} y}} (kt) = \frac{kt}{{k t + \frac{{\left\| {x - y} \right\|}}{{M_{n}}}}} = \frac{{kt M_{n}}}{{kt M_{n} + \left\| {x - y} \right\|}};\quad \forall t \in {\varvec{R}}_{+},\; \forall n \in {\varvec{Z}}_{0+}$$

where *M*_*n*_ = *f*(*n*)(1 + *x*)(1 + *y*). On the other hand:

$$F_{x,y} (t) = \frac{t}{{t + \left\| {x - y} \right\|}}; \quad \forall {\text{t}} \in {\varvec{R}}_{+}$$

so that the condition $F_{{T_{n} x,T_{n} y}} (kt) \ge F_{x,y} (t)$ becomes:

34 $$\frac{{k t M_{n}}}{{k t M_{n} + \left\| {x - y} \right\|}} \ge \frac{t}{{t + \left\| {x - y} \right\|}};\quad \forall {\text{t}} \in {\varvec{R}}_{+}.$$

If we denote now $q = k M_{n}$ and consider the function $g(q) = \frac{qt}{{qt + \left\| {x - y} \right\|}}$ we have:

$$\frac{dg(q)}{dq} = \frac{d}{dq}\left({\frac{qt}{{qt + \left\| {x - y} \right\|}}} \right) = \frac{{\left\| {x - y} \right\|t}}{{\left({qt + \left\| {x - y} \right\|} \right)^{2}}} \ge 0; \quad\forall t \in {\varvec{R}}_{+}$$

Therefore, *g*(*q*) is a non-decreasing function and if *q* ≥ 1 we have *g*(*q*) ≥ *g*(1). Thus, Eq. (34) holds provided that $q = k M_{n} \ge 1$, condition that can be achieved if:

$$k \ge \frac{1}{{inf\;M_{n}}} \ge \frac{1}{{M_{n}}}$$

**Fig. 1:**
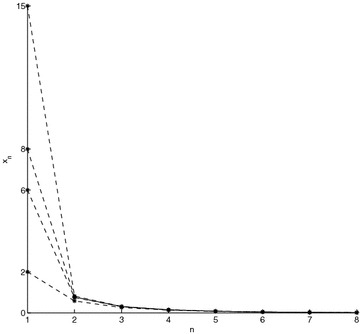
Evolution of the sequence of iterates for different initial conditions

**Fig. 2:**
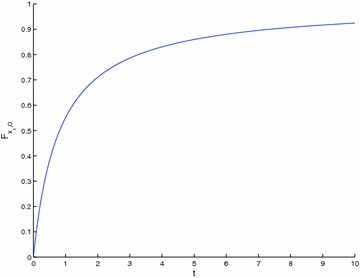
Convergence of the sequence of probability functions $F_{{x_{n},x^{*}}} (t)$ to unity. Display of $F_{{x_{1},0}} (t)$

**Fig. 3:**
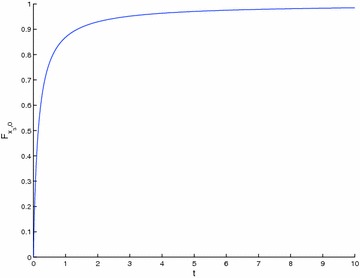
Convergence of the sequence of probability functions $F_{{x_{n},x^{*}}} (t)$ to unity. Display of $F_{{x_{3},0}} (t)$

**Fig. 4:**
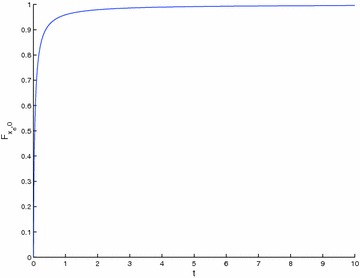
Convergence of the sequence of probability functions $F_{{x_{n},x^{*}}} (t)$ to unity. Display of $F_{{x_{6},0}} (t)$

Moreover, *inf M*_*n*_ = *inf*(*f*(*n*)(1 + *x*)(1 + *y*)) = *inf f*(*n*) = 2 since *X* = [0, +∞) and Eq. (34) holds if $k \ge \frac{1}{2}$. Therefore, we can choose $\frac{1}{2} \le k < 1$ and *T*_*n*_ is a strict *k*-contraction for every non-negative *n*. Furthermore, if we select now the dual strict comparison function $\varphi$(*t*) = 1, *T*_*n*_ is also a strict dual $\varphi$-contraction since $F_{{T_{n} x,T_{n} y}} (kt) \ge F_{x,y} (t)$ still holds when *k* = 1 (Definition 4). Therefore, Theorems 1, 3 and 6 hold and the nonlinear iterative scheme:

35 $$x_{n + 1} = \frac{{x_{n}}}{{f(n)(1 + x_{n})}}$$

converges to the unique fixed point of $T = {lim}_{n \to \infty} T_{n} = {lim}_{n \to \infty} \frac{x}{f(n)(1 + x)} = \frac{x}{2(1 + x)}$ which is x^*^ = 0. Figure 1 shows the evolution of the iterative scheme (35) for different initial conditions. It can be seen in Fig. 1 that the sequence of iterates converges to zero as predicted by Theorem 2(iii), Theorems 3 and Theorem 6. Moreover, the sequence of probability functions $F_{{x_{n},x^{*}}} (t) = F_{{x_{n},0}} (t)$ converges to unity as Figs. 2, 3 and 4 show.

## Conclusions

This paper has investigated some relevant properties of convergence of sequences built through sequences of operators which are either uniformly convergent to either a strict *k*-contractive operator, for some given real constant $k \in (0,1)$, or which are strictly *k*-contractive and point-wisely convergent to some limit operator. Those convergence properties are also reformulated for the case when either the sequence of operators or its limit are strict $\varphi$-contractions. The limits of the built convergent sequences are either the limits of the sequence of fixed points of the corresponding sequences of operators or the fixed points of the limit operator of the sequence of operators. The definitions of strict *k*-contractions and $\varphi$-contractions are given in the context of probabilistic metric spaces for the given probability density function. A numerical illustrative example is proposed and discussed in detail.
